# System for Measuring Conditional Amplitude, Phase, or Time Distributions of Pulsating Phenomena

**DOI:** 10.6028/jres.097.030

**Published:** 1992

**Authors:** Richard J. Van Brunt, Eric W. Cernyar

**Affiliations:** National Institute of Standards and Technology, Gaithersburg, MD 20899

**Keywords:** amplitude distributions, conditional distributions, electronic circuits, memory propagation, multichannel analyzer, partial discharges, phase distributions, pulsating phenomena, stochastic analyzer, time separation distributions, Trichel pulses

## Abstract

A detailed description is given of an electronic stochastic analyzer for use with direct “real-time” measurements of the conditional distributions needed for a complete stochastic characterization of pulsating phenomena that can be represented as random point processes. The measurement system described here is designed to reveal and quantify effects of pulse-to-pulse or phase-to-phase memory propagation. The unraveling of memory effects is required so that the physical basis for observed statistical properties of pulsating phenomena can be understood. The individual unique circuit components that comprise the system and the combinations of these components for various measurements, are thoroughly documented. The system has been applied to the measurement of pulsating partial discharges generated by applying alternating or constant voltage to a discharge gap. Examples are shown of data obtained for conditional and unconditional amplitude, time interval, and phase-of-occurrence distributions of partial-discharge pulses. The results unequivocally show the existence of significant memory effects as indicated, for example, by the observations that the most probable amplitudes and phases-of-occurrence of discharge pulses depend on the amplitudes and/or phases of the preceding pulses. Sources of error and fundamental limitations of the present measurement approach are analyzed. Possible extensions of the method are also discussed.

## 1. Introduction

There are many types of naturally occurring pulsating phenomena that have statistical properties which have not yet been adequately explained. Included in this category of phenomena are certain types of nerve impulses, pulsating fluid flow and droplet formation, bursts of electromagnetic radiation from extraterrestrial sources, geological disturbances such as earth tremors, and pulsating electrical discharges specifically considered in this work. These phenomena may exhibit complex chaotic behavior manifested by an apparent high degree of randomness in the time of occurrence and magnitude of the impulse events. For some phenomena, the complexity of the impulse behavior may, in part, be a consequence of memory propagation between successive events. In developing a better understanding of the physical bases for pulsating phenomena, it is essential to assess the effects of memory propagation.

In the case of pulsating partial-discharge phenomena, it has already been shown that effects of memory propagation are significant [1–4]. Partialdischarge (PD) phenomena are of special interest because they are types of localized electrical discharges that occur at defect sites in electrical insulation. Partial discharges are often the precursors to insulation failure and represent undesirable electrical noise sources under some conditions. The detection of PD pulses has been used to assess insulation performance and integrity [5]. It is also known [6, 7] that PD phenomena exhibit stochastic properties that depend on the nature of the defect site such as characterized by the types of materials present as well as their geometrical configuration. Partial discharges also produce physical or chemical changes in the characteristics of the defect sites (PD – induced aging) that in turn produce changes in the stochastic behavior of the discharge [8–11].

Efforts have been underway in numerous laboratories to quantify statistically PD patterns using computer-assisted measurement and analysis techniques [12–22]. The incentive for this work has been the development of so called “smart” PD detectors that employ pattern recognition to help identify the type of defect at which the PD occurs, e.g., to distinguish between a cavity in solid insulation and a metal particle in liquid or gaseous insulation. Unfortunately, progress in the development of reliable automated methods for PD pattern recognition has been hampered by a failure to understand the physical mechanisms that determine the stochastic properties of PD phenomena. In general, present computer assisted PD-measurement systems simply do not provide enough refined information about the stochastic properties of PD pulses for a meaningful analysis.

The purpose of the present work is to describe a real-time stochastic analyzer that can be used to quantify the stochastic behavior of a train of electrical pulses that may or may not be correlated with a periodic time varying excitation source, e.g., a sinusoidal voltage. The instrument described here is an extended version of one that was used to investigate the stochastic behavior of pulsating negative-corona discharges generated by applying a constant voltage to a point-plane electrode gap [1, 24, 25]. In addition to the conditional pulse-amplitude and time-separation distributions that could be measured with the previous system, the present system also allows measurement of a set of phase-restricted pulse-amplitude and phase-of-occurrence distributions. This latter capability makes the instrument suitable for investigating the stochastic behavior of partial discharges generated using alternating voltages. The data acquired from this system provide immediate determinations of the existence of pulse-to-pulse or phase-to-phase memory propagation effects.

The measurement system described here can be thought of as a type of electronic filter that is inserted between the impulse source and a computer-driven multichannel analyzer (MCA) in which data on the desired conditional or unconditional pulse distributions are accumulated. The unique features of the circuitry of this filter are documented here in enough detail to allow replication. The present system design incorporates standard commercially available nuclear-instrumentation components, where possible, such as time-to-amplitude converters and linear pulse amplifiers. Although the present system can be employed to investigate any type of pulsating phenomenon that can be converted to electrical signals, it was designed primarily for the measurement of relatively stationary PD-pulse phenomena generated by a constant or low-frequency alternating voltage. The system may not be well suited for investigations of impulses that have repetition rates much greater or less than the PD-phenomena considered here; and it will not perform well for phenomena that exhibit highly nonstationary behavior, i.e., phenomena for which the stochastic properties change rapidly with time.

The range of phenomena to which the present system can be applied is considered and the system’s inherent limitations and sources of error are analyzed. Extensions of the technique and alternative approaches that rely primarily on analysis using computer software are discussed. Examples are presented of results obtained for partial discharges generated in a point-to-solid dielectric electrode gap.

## 2. Definitions

In this section we introduce the parameters that define the types of stochastic processes which can be investigated with the electronic measurement system described here. We also define the various conditional and unconditional distributions that are measured with this system and indicate how the measured distributions can be used to gain insight into the physical bases for the process under investigation.

### 2.1 Random Point Processes

The types of pulsating phenomena to be considered here are those that can be represented by a *marked random point process* as defined by Snyder [26]. In order to represent the phenomenon as a point process, the pulses must occur at discrete times that can be readily defined. In the case of a periodic time-varying excitation, the events of interest must occur at discrete phases. This requires that an occurrence time (or phase) can be meaningfully associated with a particular property of the pulse such as its amplitude. Difficulties can be encountered in satisfying the criterion for a point process if, for example, there is significant variability in the shapes of the pulses or if there is the possibility that successive pulses can overlap or otherwise become indistinguishable. Ideally there should be a reasonable uniformity in pulse shapes and the mean spacing between pulses should be much greater than the pulse widths. The types of pulsating partial-discharge phenomena to which the present measurement system have been applied generally satisfy the requirements for a point process.

It is also assumed that the point process can be *marked* by some property of the pulse such as its amplitude, width, shape parameters, or area under the pulse. In order to consider the mark as a property of the pulse measured with the present system, it is necessary that the mark be converted to an electrical signal with a voltage that is proportional to the “size” of the mark. For reasons previously discussed [1,27], the partial-discharge pulse amplitude has been selected here as an appropriate mark which is a measure of discharge magnitude. Since PD can be detected by different methods, e.g., optical, acoustical, and electrical [5], it is necessary to convert the observed response to an electrical signal as is normally done for purposes of recording data.

If the occurrence of the pulsating phenomenon is correlated with an externally controlled time-varying excitation process such as a chopped light beam or, as is sometimes the case for PD pulses, a sinusoidal voltage, then it may be more convenient to specify the time of pulse occurrence relative to the times of the external excitation processes. For PD-pulses generated with an alternating sinusoidal voltage, it is desirable to consider the *phase-of-occurrence* of a pulse as defined by the phase of the corresponding applied voltage at the time of PD-initiation.

The point processes under consideration here are assumed to be *random* in the sense that both the times-of-occurrence and the marks can exhibit statistical variability, e.g., it is not possible to predict precisely when a given pulse will occur or what its amplitude will be. For processes excited by a well-defined controllable periodic source, it is also possible to define point processes that are fixed in time or phase but exhibit statistical variability in the mark. Such a process might be, for example, the sum of the areas under all PD pulses that occur in a specified phase interval of the applied voltage. The sum could be recorded at a fixed phase immediately following the time lapse of the phase interval. The measurement system described in this work allows determination of such phase-restricted sums of pulse areas or amplitudes.

### 2.2 Measurable Quantities for a PD Process (Random Variables)

The type of marked random point process under consideration here is a stochastic process specified by a countable set of discrete *random variables* of which time-of-occurrence (or equivalently phase-of-occurrence) is one of the variables. In this section we define the sets of random variables that apply to the measurement of pulsating partial discharges generated either with a constant applied voltage (dc) or an alternating (sinusoidal) applied voltage (ac).

#### 2.2.1 Random Variables for a dc-Excited PD Process

**Fig. 1:**
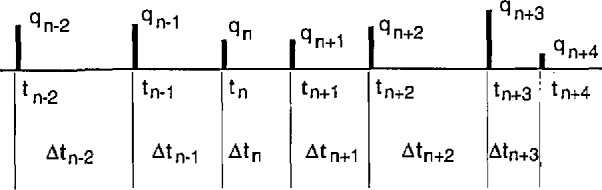
Diagrammatic representation of a marked random point process. Shown are pulses “marked” with amplitudes *q_j_, j =n* − 2,*n*−1,…, that occur at discrete times *t_j_* with corresponding time separations Δ*t_j_.*

A diagrammatic representation of a degenerated PD process is shown in Fig. 1. As previously discussed [1,24,25], this process can be specified by the finite set {*q_i_, t_i_*}*_n_*, *i* = 1,2,…,*n* where *q_i_* is the amplitude of the *i*th PD pulse (usually expressed in units of picocoulombs) and *t_i_* is the time at which this pulse occurs. The measurement system described here records time separations between successive PD events rather than actual occurrence times. It is therefore more convenient to specify the process in terms of the set of random variables {*q*_1_, *q_i_*, Δ*t_i_*_−1_}*_n_*, *i* =2,…, *n*, where Δ*t_i_*_−1_*=t_i_* − *t_i_*_−1_ is the time separation be-tween the (*i*−1)th and *i*th events. To satisfy the requirements for a point process, it is desirable that the mean duration of the PD events, as measured, for example, by the pulse widths, *δt_i_*, be much smaller than the mean time separation between successive events, i.e., (Δ*t_i_*) ≫ (*δt_i_*) for all values of *i.* If all time intervals are recorded, the time-of-occurrence of any pulse can simply be determined from the sum

$$t_{i}=\sum_{j=1}^{i-1}\Delta t_{j}.$$ (1)

As will be seen from the discussion below, data on the time separations between events are needed to assess pulse-to-pulse memory propagation effects. If memory effects are important, the random variables associated with the amplitudes and time separations of successive pulses are not independent, e.g., the amplitude of any given pulse can depend on the time separation between that pulse and the previous pulse.

#### 2.2.2 Random Variables for an ac-Excited PD Process

**Fig. 2:**
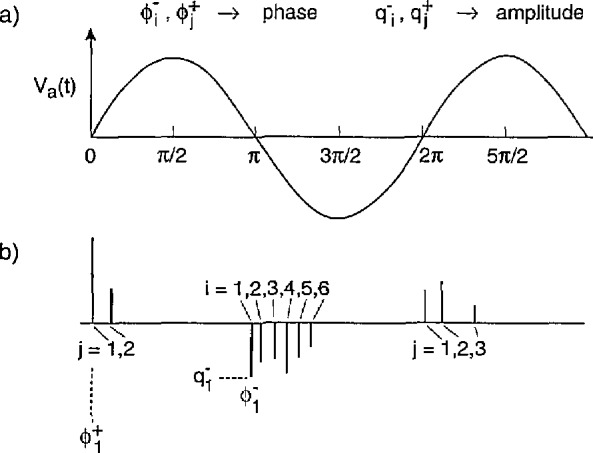
Diagrammatic representation of an ac-excited partial discharge process: a) sinusoidal excitation voltage, b) phase-correlated PD pulses.

If the PD process is generated with an alternating voltage, it becomes more convenient to specify the *phase-of-occurrence* of the PD pulse rather than the time-of-occurrence. An example of an ac-generated PD process is shown by the diagram in Figs. 2a and 2b. The excitation voltage indicated in Fig. 2a is assumed to be sinusoidal and is given by

$$V_{a}(t)=V_{0}\sin(\omega t)=V_{0}\sin[\phi (t)],$$ (2)

where *ω*/2π is the frequency, *ϕ(t) = ωt* the phase, and *V*_0_ is the amplitude. The individual PD events are specified by the set of random variables ${\left\{q_{ij}^{\pm },\phi_{ij}^{\pm }\right\}}_{n,m}$, *i* = 1,2,…,*n*; *j* = 1,2,…,*m* where $q_{ij}^{+}$ and $q_{ij}^{-}$ are the amplitudes of the *i*th pulses to appear respectively in the *j*th positive and negative half-cycles of the applied voltage and $\phi_{ij}^{+}$ and $\phi_{ij}^{-}$ *are* their corresponding phases-of-occurrence. The phases $\phi_{ij}^{\pm }$ are restricted by definition to lie within the interval (0, 2*π*) for arbitrary; and are thus related the phase at time *t* by $\phi (t)=\phi_{ij}^{\pm }+2\pi (j-1)$.

The amplitudes for $q_{ij}^{+}$ and $q_{ij}^{-}$ are observed to be of opposite signs as indicated in Fig. 2b. In some cases, as previously explained [3, 4], the occurrence of positive and negative PD pulses may be phase shifted relative to the positive and negative half-cycles, e.g., it may be possible for negative pulses to occur before the zero-crossing where the voltage is still positive. This is phyiscally a consequence of a fluctuating phase lag in the local electric-field strength at the discharge site. The phase shift will be denoted here by *δϕ*, and is arbitrarily adjusted to a value such that

$$\phi_{ij}^{+}\epsilon (-\delta \phi ,\pi -\delta \phi ),\phi_{ij}^{-}\epsilon (\pi -\delta \phi ,2\pi -\delta \phi )$$ (3)

for all values of *i* and *j*.

As in the case of dc-generated PD pulses, it may also be useful to specify the phase differences between successive events within a half-cycle. These phase differences will be denoted by $\Delta \phi_{i,i-1,j}^{\pm }\equiv \phi_{i,j}^{\pm }-\phi_{i-1,j}^{\pm }$ where *i* ⩾ 2.

For some types of ac-generated PD processes, especially those that occur in the presence of solid dielectric surfaces, it is valuable in assessing phase-to-phase memory propagation effects to know the accumulated PD charge associated with each half-cycle as defined by

$$Q_{j}^{\pm }=\sum_{i}q_{ij}^{\pm },$$ (4)

where the summation is over amplitudes of all pulses that occur within a given half-cycle as specified by their phases-of-occurrence defined in Eq. (3).

It is possible with the system described here, and sometimes necessary, to record the number of individual voltage cycles. This is necessary if an assessment is to be made of memory propagation that extends back beyond the previous cycle. For most types of measurements described here however, this information is not recorded. If no attempt is made to record the number of a given cycle, then the subscript; can be dropped from the specification of the random variables that define the stochastic process for ac-generated PD. In this case, the appropriate designation of the random variables is $q_{i}^{\pm }$, $\phi_{i}^{\pm }$, $\Delta \phi_{i,i-1}^{\pm }$, and *Q*^±^. A failure to include the subscript on the variable *Q*^±^ will imply by default that the sum given by Eq. (4) applies to the half-cycle immediately preceding that in which the variables $q_{i}^{\pm }$, $\phi_{i}^{\pm }$, are measured.

In performing the measurement of an ac-generated PD process, it is assumed that the excitation voltage given by Eq. (2) is instrumentally filtered out or otherwise subtracted from the PD signals. This is necessary to ensure that the recorded pulse amplitude is a true measure of the discharge intensity.

### 2.3 Measurable Conditional and Unconditional Distributions

#### 2.3.1 Unconditional Distributions

Because the variables such as pulse amplitude and phase- of-occurrence that describe the pulsating phenomenon (PD process) of interest are random, they can only be specified quantitatively in terms of statistical probability distributions. The unconditional probability distribution *p_ξ_*(*x*) (sometimes referred to as the probability density function [28]) for a random variable, *ξ*, is defined such that *p_ξ_*(*x*) d*x* is the probability that *ξ* will assume a value that lies in the interval *x* to *x +* d*x.* Here *ξ* can be any of the random variables that were defined in the previous section.

Consistent with our earlier work [1,24], we shall adopt the abbreviated notation for distribution functions whereby *p_ξ_*(*x*) d*x* is replaced by *p*_0_(*ξ*) d*ξ*. Thus, for example, *p*_0_(*q_n_*) d*q_n_* is the probability that the *n*th pulse has amplitude between *q_n_* and *q_n_ +* d*q_n_*. There is no ambiguity in this notation if it is understood that the symbol used to designate the *value* of a random variable is the same as that used to define the variable.

The distribution *p*_0_(*ξ*) is *unconditional* in the sense that it gives the probability that the random variable will have a particular value independent of the past history of the process, e.g., independent of values for random variables associated with previous events. The random variables, as defined here, correspond to particular discrete events in time associated with the random point process, i.e., *ξ = q_n_*, Δ*t_n_*_−1_, *ϕ_n_*, where the subscript *n* is assigned to the *n* th event. In cases where the events are not actually counted by the measurement process, the distributions such as *p*_0_(*q_n_*) and *p*_0_(Δ*t_n_*) are assumed to apply to arbitrary *n.* If events are counted relative to a specified time, then *n* is assigned a value, e.g., *p*_0_(*q*_2_^+^) is the amplitude distribution of the second pulse to appear in an arbitrary positive half-cycle of the excitation voltage.

#### 2.3.2 Conditional Distributions

If memory effects are important in a pulsating process, then the probabilities that the random variables associated with a particular event will have specific values depend in general on the values for these variables that were assumed by previous events. The probability that the *j*th PD pulse will have values for amplitude and time separation that lie in the ranges *q_j_* to *q_j_ +* d*q_j_* and Δ*t_j_*_−1_ to Δ*t_j_*_−1_ + d(Δ*t_j_*_−1_)can depend on values of all previous *q_i_* and Δ*t_i_*_−1_ where *i* < *j.* The existence of memory effects can be established by the measurement of *conditional* probability distributions. The system to be de- scribed here allows measurement of a set of conditional distributions for such variables as pulse amplitude and phase-of-occurrence.

The conditional distribution *p*_1_(*q_j_*|Δ*t_j_*_−1_) is defined such that *p*_1_(*q_j_*|Δ*t_j_*_−1_) is the probability that the *j*th pulse has an amplitude in the range *q_j_* to *q_j_ +* d*q_j_*, if its time separation from the previous pulse has a *fixed* value Δ*t_j_*_−1_. With the system described here, it is also possible to measure higher order conditional distributions such as *p*_2_(*q_j_*|Δ*t_j_*_−1_, Δ*t_j_*_−2_), *where p*_2_(*q_j_*|Δ*t_j_*_−1_, Δ*t_j_*_−2_)d*q_j_* is the probability that the amplitude of the *j*th pulse is in the range *q_j_* to *q_j_ +* d*q_j_ if both* Δ*t_j_*_−1_ and Δ*t_j_*_−2_ are fixed. Lists of the conditional distributions that can be measured for dc and ac-generated PD pulses are given respectively in Tables 1 and 2. Determination of the conditional distributions such as *p*_1_(*q_j_*|Δ*t_j_*_−1_) provides an indication of the dependence of the random variable *q_j_* on Δ*t_j_*_−1_. If memory effects are important, then the probability that *q_j_* will assume a particular value can depend on the value chosen for Δ*t_j_*_−1_. In this case, the conditional distribution, *p*_1_(*q_j_*|Δ*t_j_*_−1_), will not equal the unconditional distribution, *p*_0_(*q_j_*), for at least some allowed values of Δ*t_j_*_−1_.

A quantitative assessment of memory propagation can be made from calculation of *expectation values* using measured conditional distributions. For example, the expectation value for the phase-of-occurrence of the third pulse in a negative half-cycle of the applied excitation voltage conditioned on a fixed value for the sum of all PD pulse amplitudes in the previous positive half-cycle is defined by

$$\langle \phi_{3}^{-}(Q^{+})\rangle =\int_{\pi -\delta \phi }^{2\pi -\delta \phi }\phi_{3}^{-}p_{1}(\phi_{3}^{-}|Q^{+})d\phi_{3}^{-},$$ (5)

where it is assumed that $\phi_{3}^{-}$ must be confined to the interval defined by Eq. (3). In general,

$$\langle \xi_{i}({\left\{a_{k}\right\}}_{n})\rangle =\int_{R}\xi_{i}p_{n}(\xi_{i}|{\left\{a_{k}\right\}}_{n})d\xi_{i},$$ (6)

where *ξ_i_*, is any random variable associated with the *i*th pulse and {*a_k_*}*_n_* is a set of fixed values for *n* random variables associated with one or more pulses that occurred at earlier times. The integral in Eq. (6) is over all allowed values of *ξ_i_* that are assumed to lie within a range *R*, i.e., *ξ_i_*∊ *R.*

If memory effects are important, the value of 〈*ξ_i_*({*a_k_*}*_n_*)〉 will change as one or more of the values *a*_k_ are changed. If the value of 〈*ξ_i_*({*a_k_*}*_n_*)〉 increases as *a_l_* ∊ {*a_k_*}*_n_* increases within a particular range (*a_l_* ∊ *A_l_*), then *ξ_i_*, is said to be *positively dependent* on *a_l_* in that range for fixed values of *a_k_*(*k* ≠ *l*). Consistent with our earlier notation [1], this dependence is denoted by (*a_l_* ↑ ⇒ *ξ_i_* ↑, {*a_k_*, *k* ≠ *l*}) when *a_l_* ∊ *A_l_.* Likewise *ξ_i_* can be *negatively dependent* on a different variable or on *a_l_* in a different range *A*′*_l_*. In this case, the negative dependence is denoted by (*a_l_* ↑ ⇒ *ξ_i_* ↓, {*a_k_*, *k* ≠ *l*}) when *a_l_* ∊ *A*′*_l_* The dependence of the expectation values for random variables on the values of random variables associated with prior events can often be predicted from physical models of the process as has been done for the case of negative corona (Trichel) type partial- discharge pulses [1].

If memory effects are important in the pulsating phenomenon, then the various distributions listed in Tables 1 and 2 are not necessarily independent. It can be shown, for example, from the law of probabilities that the distributions *p*_0_(*q_j_*), *p*_0_(Δ*t_j_*_−1_), and *p*_1_(*q_j_*|Δ*t_j_*_−1_) are related by the integral expression

$$p_{0}(q_{j})=\int_{0}^{\infty }p_{0}(\Delta t_{j-1})p_{1}(q_{j}|\Delta t_{j-1})d(\Delta t_{j-1}).$$ (7)

Similarly the distributions $p_{0}(\phi_{i}^{\pm })$, $p_{0}(\phi^{\pm })$ and $p_{1}(\phi_{i}^{\pm }|Q^{\mp })$ are related by

$$p_{0}(\phi_{i}^{\pm })=\int_{0}^{\infty }p_{0}(Q^{\mp })p_{1}(\phi_{i}^{\pm }+Q^{\mp })dQ^{\mp }.$$ (8)

It has previously been shown [1] that there may be many other integral expressions that connect the different conditional and unconditional measurable distributions. Equations (7) and (8) can be used to check the consistency among the various measured distributions. For example, if data are obtained on the three distributions *p*_0_(*q_i_*), *p*_0_(Δ*t_j_*_−1_) and *p*_1_(*q_j_*|Δ*t_j_*_−1_), then one should, if possible, verify that they satisfy Eq. (7). There may be some cases, however, where it is not possible to obtain enough data at high enough resolution to perform this analysis.

In the process of measuring a conditional distribution it is generally not possible to select a single value for the “fixed” variable. This variable can only be specified experimentally to lie within a finite window. In the case of the distribution $p_{1}(\phi_{i}^{-}|Q^{+})$, for example, one really measures an approximation to this distribution given by

$$P_{1}(\phi_{i}^{-}|Q^{+})=\int_{Q^{+}-\delta Q^{+}}^{Q^{+}+\delta Q^{+}}p_{0}({Q^{\prime }}^{+})p_{1}(\phi_{i}^{-}|{Q^{\prime }}^{+})d{Q^{\prime }}^{+}$$ (9)

where Q′^+^ is defined by the measurement to lie within the window corresponding to the interval (*Q^+^* − *δQ^+^*, *Q^+^ + δQ^+^*). The measured conditional distribution approaches the “true” conditional distribution as the window is made smaller, e.g.,

$$P_{1}(\phi_{i}^{-}|Q^{+})=\underset{\delta Q^{+}\to 0}{\lim}p_{1}(\phi_{i}^{-}|Q^{+}\pm \delta Q^{+}).$$

The errors associated with finite window size have previously been noted [24] and will be discussed again later in this work.

All conditional distributions satisfy the normalization requirement

$$\int_{R}p_{n}(\xi_{i}|{\left\{a_{k}\right\}}_{n})d\xi_{i}=1.$$ (10)

Measured data for conditional distributions are generally normalized according to this requirement by numerical integration. In some cases, it may be convenient for display purposes to normalize to the maximum of the distribution.

## 3. Measurement System

In this section we describe the general features of the system for measuring the conditional and unconditional distributions listed in Tables 1 and 2. The system can be configured to investigate either a continuous train of pulses produced by a constant excitation process, e.g., dc-generated PD or pulses generated by periodic, time-varying process, e.g., ac-generated PD. Thus, the system is an extended version of that previously described for measurement of dc-generated PD [1, 24, 25], and, in fact, includes all of the features of the earlier system. We shall treat the ac and dc measurement configurations separately even though they both utilize some of the same individual circuit components.

### 3.1 Configurations for a Continuous Excitation Process (de-Generated PD)

**Fig. 3:**
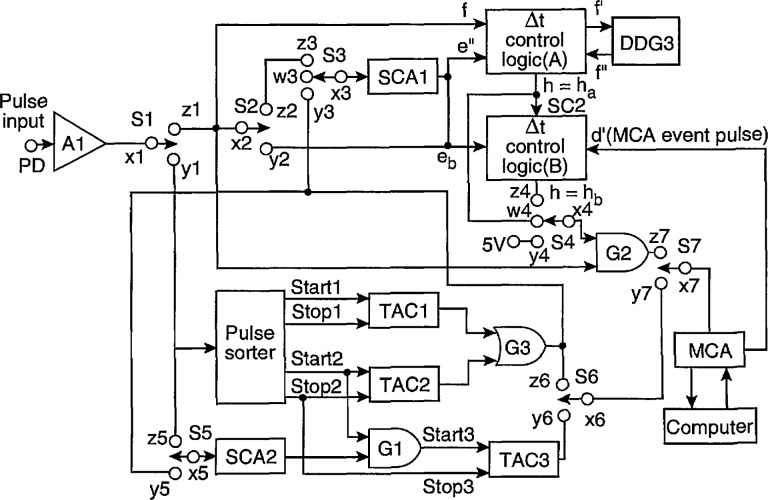
System for measuring unconditional and conditional pulse-amplitude and time-separation distributions for a continuous or dc excited point process such as shown in Fig. 1. A = amplifier, DDG = digital delay generator, TAC = time-to-amplitude converter, SCA = single-channel analyzer, MCA = multichannel analyzer, G = gate, S = switch.

The configurations of the electronic system used to measure the distributions listed in Table 1 for a dc-generated PD process have been described previously [1, 24, 25], a block diagram indicating the circuit components utilized for this case is shown in Fig. 3. The configurations of the components that are required for the measurement of each distribution are specified by the various switch configurations listed in Table 3 where *S*1–*S*7 are the switches designated in Fig. 3 and the notation *a*1 *= b*1 implies, for example, that position *a*1 of *S*1 is connected to position *b*1 of *S*1.

The system indicated in Fig. 3 differs from that used previously mainly in the design of the individual circuit components that will be described in the next section. The most significant changes have been in the design of the Δ*t* control logic circuits (parts A and B). Although their basic function and operation are the same as previously described [1,24], changes were made that reduce errors, improve performance, and eliminate redundancies. Some of these changes have already been utilized in the investigation of Trichel pulses [1], but have never been documented in detail.

The pulse sorter circuit that drives the time-to-amplitude converters (TACs) and the analog gate G3 that accepts the outputs of the TACs remain unchanged. The TACs, digital-delay generator (DDG) and the 256-channel multichannel analyzer (MCA) are commercially available instruments. The commercial single-channel analyzers (SCAs) have been replaced with a circuit to be described in the next section.

The operating principles of the system shown in Fig. 3 have been discussed already in previous publications. This configuration allows the recording of either pulse amplitudes or pulse time separations with a computer controlled MCA. The MCA employs a fast analog-to-digital converter to digitize the voltage amplitude of pulses received at its input provided the amplitude is above a preset discrimination level. If the MCA is set by switch S7 to measure pulse amplitudes, then the input signals are derived from a linear pulse amplifier, Al, after passing through the analog gate G2. This gate is a built-in feature of the MCA. If time intervals are recorded, then the MCA input is derived from the output of one or more TACs. The output of the TAC is a narrow pulse with an amplitude that is directly proportional to the time between the start and stop pulses that are applied respectively to the “start” and “stop” inputs. For the measurement of the unconditional amplitude and time separation distributions (*p*_0_(*q_j_*) and *p*_0_(Δ*t_j_*)), the gate G2 is held continuously open by positioning switch S4 such that x4=y4.

The measurement of conditional pulse-amplitude distributions requires the use of the Δ*t* control logic circuits and the digital-delay generator DDG3. For the measurement of the first-order conditional distribution *p*_1_(*q_j_*|Δ*t_j_*_−1_), the gate, G2, to the MCA is enabled by the output of the Δ*t* control logic (part A) for a time interval Δ*t_j_*_−1_ ± *δ*(Δ*t_j_*_−1_) after the *j* − 1 th pulse, provided no pulse from Al occurs within the interval 2*δ*(Δ*t_j_*_−1_) starting from the *j* − 1 th pulse. The time delay, Δ*t_j_*_−1_ and window ± *δ*(Δ*t_j_*_−1_) are determined respectively by the settings of the DDG3 delay and pulse width. The *j*−1 th pulse essentially triggers DDG3 after passing through the Δ*t* control logic (part A). The DDG3 then returns a 5 V logic pulse of preset delay and width which is in turn transferred to G2 if no other pulses have appeared at the input *f*. Details of the Δ*t* control logic circuit operation are given in the next section.

The measurement of the distributions *p*_1_(*q_j_*|Δ*t_j_*_−i_), where *i* > 1 and Δ*t_j_*_−i_ is the interval between the *j* − *i* th and *j* − *i* + 1 th events, requires the use of both parts A and B of the Δ*t* control logic circuit. The value of *i* is determined by a selectable pulse counter inherent to the Δ*t* control logic (part B) as described in the next section. The time interval Δ*t_j_*_−i_ is selected by part A of the Δ*t* control logic in conjunction with DDG3 as in the case of the *p*_1_(*q_j_*|Δ*t_j_*_−1_) measurement. The output of part A is then used to trigger part B. If a pulse appears at the input e_b_ of part B within the time interval Δ*t_j_*_−1_ ± *δ*(Δ*t_j_*_−1_), then the gate G2 to the MCA is enabled either immediately for measurement of *p*_1_(*q_j_*|Δ*t_j_*_−2_) or after *i* − 2 pulses have been counted for measurement *of p*_1_(*q_j_*|Δ*t_j_*_−i_), *i* > 2. The next pulse to appear after G2 is enabled will be recorded by the MCA which then returns an “event pulse” to reset part B of the Δ*t* control logic. The next input pulse time interval to lie within the range Δ*t_j_*_−_*_i_* ± *δ*(Δ*t_j_*_−_*_i_*) will start the process over again.

The second-order conditional pulse-amplitude distributions, *p*_2_(*q_j_*|Δ*t_j_*_−1_, *q_j_*_−1_) and *p*_2_(*q_j_*|Δ*t_j_*_−1_, Δ*t_j_*_−2_) can be measured by using a single-channel analyzer, SCAl, the output of which is connected to input e″ of the Δ*t* control logic circuit (Part A), Depending on the position of switch SI, SCAl receives a pulse either directly from amplifier Al or from the output of TAC1 for measurement of *p*_2_(*q_j_*|Δ*t_j_*_−1_, *q_j_*_−1_) and *p*_2_(*q_j_*|Δ*t_j_*_−1_, Δ*t_j_*_−2_) respectively. In the first case, the Δ*t* control logic and subsequently DDG3 are only triggered if the amplitude of *q_j_*_−1_ lies within a narrow range selected by SCAl. In the second case, it is triggered only if the output pulse of TAC1, the amplitude of which is proportional to Δ*t_j_*_−2_, lies within a narrow range corresponding to Δ*t_j_*_−2_ ± *δ*(Δ*t_j_*_−2_) as selected again by SCAl.

The first-order distribution *p*_1_(*q_j_*|*q_j_*_−1_) can be measured using the configuration for measurement *of p*_2_(*q_j_*|Δ*t_j_*_−1_, *q_j_*_−1_) and selecting the time window *δ*(Δ*t_j_*_−1_) from DDG3 to be large compared to the mean time separation between pulses, i.e., *δ*(Δ*t_j_*_−1_) ≫ (Δ*t_j_*_−1_). Although it is possible to mea- sure directly other types of conditional amplitude distributions with this system [24] such as *p*_1_(*q_j_*|Δ*t_j_*_−1_+Δ*t_j_*_−1_), these are derivable from the distributions listed in Table 1, hence are considered difficult to interpret and less useful in reveal- ing stochastic properties of the process.

Measurement of the unconditional time-separation distribution, *p*_0_(Δ*t_j_*), requires use of two time-to-amplitude converters (TAC1 and TAC2) connected to a pulse sorter. As previously shown, [24] this arrangement allows measurement of all successive time separations if all the time separations are greater than the TAC reset time. The reset time for the TACs used in the present measurement system is 50 μs. Failure to sample all time separations can lead to errors in the measurement *of p*_0_(Δ*t_j_*) under some conditions as will be discussed later [24].

Measurement of the conditional time-separation distributions involves use of the single-channel analyzer SCA2 that is either connected at S5 directly to Al for measurement of *p*_1_(Δ*t_j_|q_j_*) or to the output of TAC1 for measurement of *p*_1_(Δ*t_j_*_−1_|Δ*t_j_*_−1_). The output of SCA2 enables gate G1 for measurement of Δ*t_j_* with TAC3 provided either *q_j_* or Δ*t_j_*_−1_ lie within the windows selected by SCA2. The gate G1 is actually a built-in feature of the time-to-amplitude converter circuit used in the present system.

### 3.2 Configuration for a Periodic Time-Varying Excitation Process (ac-Generated PD)]

**Fig. 4:**
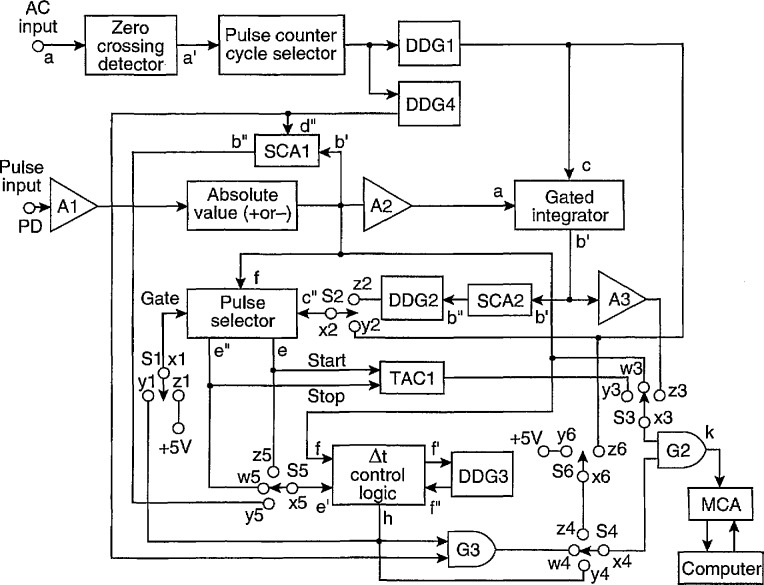
System for measuring unconditional and conditional pulse-amplitude, amplitude-sum, and phase-of-occurrence distributions for a point process excited by an alternating voltage. The individual circuit units are defined as in Fig. 3.

A diagram of the system configuration used for measurement of the distributions listed in Table 2 is shown in Fig. 4. Although it is assumed here for convenience and simplicity that the excitation process for the observed pulses is sinusoidal as given by Eq. (2), this is not a requirement for the measurement method. It is only required that the excitation process have a well defined periodicity so that phase position and intervals can be meaningfully specified. Thus, excitation processes that can be represented by a Fourier expansion are also acceptable, e.g., voltages of the form

$$V_{a}(t)=C_{0}+\sum_{n=1}^{\infty }A_{n}\cos(n\omega t)+\sum_{n=1}^{\infty }B_{n}\sin(n\omega t),$$ (11)

where *C*_0_ is a constant and *A_n_* and *B_n_* are the usual Fourier expansion coefficients.

One of the major differences between the measurement system shown in Fig. 3 and that shown in Fig. 4 is that, in the latter configuration, the measurement of pulse occurrence times are always made relative to a fixed reference phase. This reference is provided by the output of a zero-crossing detector similar to that used in our earlier work [29] which generates a positive 5 V pulse with a width of 2 *μ*s whenever the excitation voltage changes sign from negative to positive. The output of the zero-crossing detector is fed to a pulse counter. The output of the pulse counter triggers two digital-delay generators DDG1 and DDG4 used to define the phase intervals over which measurements are made. Depending on the setting of the pulse-counter output, DDG1 and DDG4 are triggered either at the beginning of every cycle of the excitation voltage or at the beginning of every *n* th cycle, where *n* is an integer greater than 1.

The MCA, TAG, gate G1, Δ*t* control logic (part A), SCAs and DDG3 are the same components used for the system configuration shown in Fig. 3. The other digital-delay generators DDG1, DDG2, and DDG4 are essentially identical in their operating characteristics to DDG3. The gated integrator and pulse selector are specifically designed for the system configuration shown in Fig. 4 and are described in the next section. The absolute-value selector circuit is similar in design to a circuit used for previous PD measurements [30]. It provides a positive pulse to amplifier A2 independent of the sign of the input pulse. It can be operated to select either positive input pulses, negative input pulses, or pulses of both signs. This feature is needed because ac-generated PD pulses are either positive or negative depending on the half-cycle of the applied voltage in which they occur. The amplifier A2 is a commercial linear pulse amplifier that has a constant adjustable dc offset at the output. It delivers a rectangular negative pulse to the gated integrator with a constant width of 2 *μ*s and with an amplitude proportional to the peak amplitude of the input pulse. It has output characteristics required for proper operation of the integrator. Shown in Tables 4 and 5 are the combinations of switch connections for S1–S5 in Fig. 4 that are required to configure the system for measurement of the various conditional and unconditional distributions given in Table 2.

**Fig. 5:**
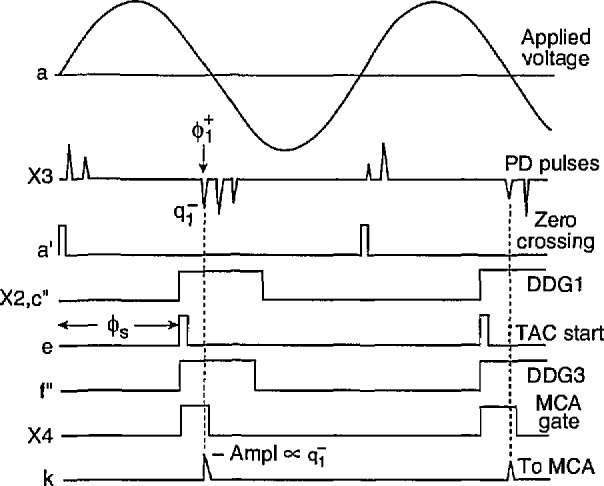
Fulse timing diagram for indicated signal locations in the system configuration (Fig. 4) for measurement of the amplitude distribution $p_{0}(q_{1}^{-})$, of the first pulse to appear on the negative half-cycle of the applied voltage.

**Fig. 6:**
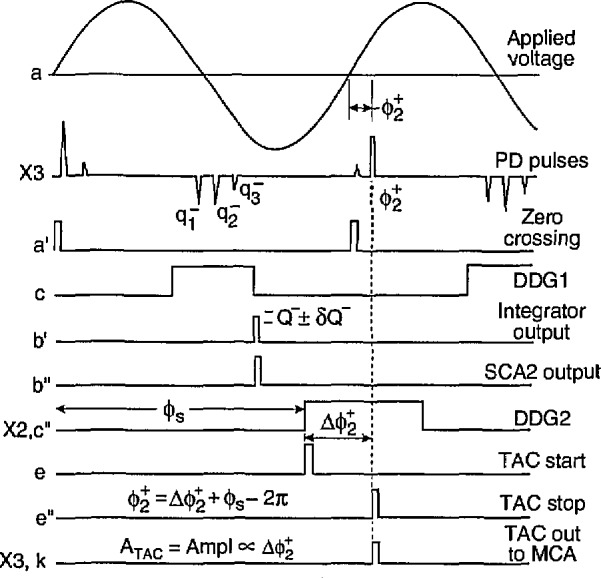
Pulse timing diagram for indicated signal locations in the system configuration (Fig. 4) for measurement of the conditional distribution $p_{1}(\phi_{2}^{+}|Q^{-})$.

**Fig. 7:**
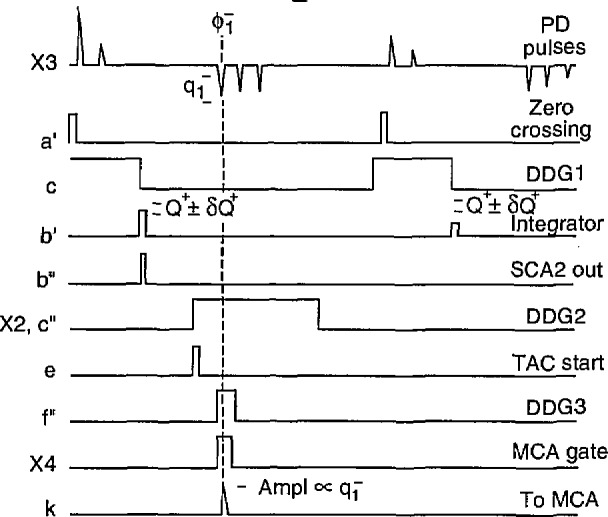
Pulse timing diagram for indicated signal locations in the system configuration (Fig. 4) for measurement of the conditional distribution $p_{2}(q_{1}^{-}|\phi_{1}^{+},Q^{+})$.

**Fig. 8:**
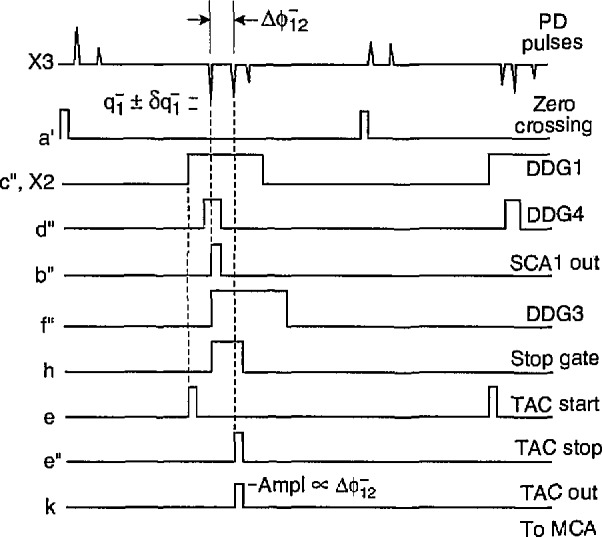
Pulse timing diagram for indicated signal locations in the system configuration (Fig. 4) for measurement of the conditional distribution $p_{2}(\phi_{2}^{-}|\phi_{1}^{+},q_{1}^{-})$.

An understanding of the system operation can be obtained with the aid of the pulse diagrams shown in Figs. 5–8. Consider first the measurement of the unconditional amplitude distribution, $p_{0}(q_{i}^{\pm })$, of the *i*th pulse in a particular half-cycle. Figure 5 shows the time sequence of signals that appear at the various indicated circuit locations in the system shown in Fig. 4 for the measurement of $p_{0}(q_{1}^{-})$, i.e., the first pulse to appear on the negative half-cycle.

The cycle selector is assumed here to be set such that every pulse *a*′ from the zero-crossing detector triggers the digital-delay generator DDG1. The output pulse from DDG1 is delayed relative to the zero-crossing pulse and its width is adjusted to encompass the entire phase region within which the negative pulses occur. This pulse is fed through switch S2 to the pulse-selector circuit which also receives pulses at location f from the pulse amplifier plus absolute value circuit. The pulse selector always generates a “start” pulse at e which is coincident with the leading edge of the DDG1 pulse at a fixed phase *ϕ*_s_. The start pulse triggers the Δ*t* control logic circuit (part A) at e′ which in turn triggers DDG3. The position and width of the DDG3 output pulse is adjusted in this case to be approximately the same as the DDG1 pulse. The output from the Δ*t* control logic opens gate G2 to the MCA. It disables this gate after the next pulse appears at the f input (in this case the first negative pulse at $\phi_{1}^{-}$). The amplitude of the first negative PD pulse is then recorded by the MCA shortly before G2 closes.

Measurement *of* $p_{0}(q_{i}^{\pm })$ for values of *i* greater than 1 requires that the Δ*t* control logic be trig- gered by the (*i* − 1)th pulse. This is achieved by connecting e′ to e through switch S5 (x5 = z5). The pulse selector will produce a pulse at e coincident in time (or equivalently in phase) with a selected pulse that occurs within the range defined by the DDG1 pulse. For example, if $p_{0}(q_{3}^{-})$ is to be measured, then the second pulse is selected to appear at e. This pulse triggers the Δ*t* control logic which in turn allows passage of the third pulse to the MCA.

The measurement of phase-of-occurrence distributions requires that the output of the time-to-amplitude converter, TAC1, be recorded by the MCA. The TAC1 circuit is triggered by the outputs from the pulse-selector circuit. The diagram in Fig. 6 shows the sequence of signals at the indicated circuit locations associated with the measurement of the conditional phase distribution $p_{1}(\phi_{2}^{+}|Q^{-})$ Again, it is assumed that the cycle selector allows every zero-crossing detector pulse to trigger DDG1. In this case, the output pulse from DDG1 is used to control a gated integrator. The width and position of the DDG1 pulse is set to encompass all possible negative pulses that could occur in the negative half-cycle.

The integrator returns a pulse at the end of the DDG1 pulse that has an amplitude proportional to the sum, $\sum_{i}q_{i}^{-}$, of the amplitudes of all negative pulses contained within the phase window defined by the DDG1 pulse. If this sum has a value that lies within the window *Q*^−^ ± *δQ*^−^ defined by the single-channel analyzer SCA2, then SCA2 triggers DDG2 which controls the pulse selector. The pulse selector produces a TAC start pulse at the leading edge of the DDG2 pulse at a fixed phase *ϕ*_s_. For the example shown in Fig. 6, the pulse selector is set to select the second positive pulse to appear in the window defined by DDG2. This pulse is used to stop TAC1 which then produces a pulse of amplitude directly proportional to $\Delta \phi_{2}^{+}=\phi_{2}^{+}-\phi_{s}$. The actual phase-of-occurrence of the second pulse relative to the zero crossing is given by

$$\phi_{2}^{+}=\phi_{s}-2\pi +\kappa A_{\text{TAC}},$$ (12)

where *A*_TAC_ is the amplitude of the TAC output pulse and *κ* is a scale factor determined from a calibration of the TAC. By this process, $\phi_{2}^{+}$ is recorded only if the sum of negative pulse amplitudes in the previous half-cycle lie within a restricted range thus yielding the conditional distribution $p_{1}(\phi_{3}^{+}|Q^{-})$.

Figure 7 shows the sequence of signals associated with the measurement of the second-order amplitude distribution, $p_{2}(q_{1}^{-}|\phi_{1}^{-},Q^{+})$. As in the case of the $p_{0}(q_{1}^{-})$ measurement indicated by the diagram in Fig. 5, this measurement requires use of both the pulse selector and Δ*t* control logic circuits. Unlike the measurement $p_{0}(q_{1}^{-})$, the amplitude, $q_{1}^{-}$, is recorded only if the phase of this pulse and the sum of amplitudes on the previous half-cycle have values that lie within restricted ranges. The phase, $\phi_{1}^{-}$, is restricted to lie within a range specified by the delay and width of the DDG3 pulse. As in the case for measurement of $p_{1}(\phi_{i}^{\pm }|Q^{\mp }),Q^{\mp }$ is specified by the SCA2 window.

There are two switch configurations that can be used to measure $p_{2}(q_{1}^{\pm }|\phi_{1}^{\pm },Q^{\mp })$. The diagram in Fig. 7 corresponds to the first set of switch connections for this distribution listed in Table 4. The second set of switch connections are required for measurement of $p_{2}(q_{i}^{\pm }|\phi_{i}^{\pm },Q^{\mp })$ if *i* ⩾ 2. The phase, $\phi_{i}^{\pm }$, in this case is restricted not by DDG3, but rather by DDG4 that controls gate G3. The gate G3 is actually a built-in feature of the Δ*t* control logic circuit (part A) as will be shown later.

The case for $p_{2}(\phi_{2}^{-}|\phi_{1}^{-},q_{1}^{-})$ is shown in Fig. 8. This measurement also requires use of both the pulse selector and Δ*t* control logic circuits. The Δ*t* control logic is used to enable a gate in the pulse selector that controls passage of the TAC stop pulse. The amplitude $q_{1}^{-}$ is restricted by the window setting for SCAl. The phase, $\phi_{1}^{-}$, is restricted by the DDG4 pulse which controls SCAl. If $q_{1}^{-}$ lies within the specified range, SCAl will produce a pulse at the end of the DDG4 pulse that triggers the Δ*t* control logic. The Δ*t* control logic uses DDG3 to open the pulse selector gate. If the next pulse to occur is indeed the second pulse, it will cause a stop pulse to be transmitted to TAC1. If the next pulse is not the second pulse, then the stop pulse output of the pulse selector will simply be disabled. Thus only the phase of $\phi_{2}^{-}$ can be recorded provided the previous pulse satisfies the specified conditions for amplitude and phase.

The measurement of unconditional amplitude sum distributions, *p*_0_(*Q*^±^), is simply obtained by transferring the output of the gated integrator directly to the MCA through amplifier A3 and gate G2. For this measurement, G2 is kept open continuously by proper positioning of S4. The amplifier A3 can be a combination of the amplifier built into the integrator and an external pulse amplifier. The gain of A3 is adjusted to give the desired range acceptable to the MCA, e.g., so that the maximum pulse amplitude is below 8 V.

The distinction between the distributions $p_{1}(q_{i}^{\pm }|\Delta \phi^{\pm })$ and $p_{1}(q_{i}^{\pm }|\phi_{i}^{\pm })$ in Table 4 is that $\Delta \phi^{\pm }$ is an *arbitrary* fixed phase window selected by DDG1 whereas $\phi_{i}^{\pm }$ is a fixed phase associated with the occurrence of *i*th pulse. The measurement of $p_{1}(q_{i}^{\pm }|\phi_{i}^{\pm })$ is like that for $p_{2}(q_{i}^{\pm }|\phi_{i}^{\pm },Q^{\mp })$ except that *Q*^∓^ is not specified. In cases where specification of *Q*^∓^ is not required, the pulse selector circuit can be controlled directly by the output pulse from DDG1 as for the case considered in Fig. 5.

The measurement $p_{1}(q_{i}^{\pm }|Q^{\mp })$ like that for $p_{2}(q_{i}^{\pm }|\phi_{i}^{\pm },Q^{\mp })$ except $\phi_{i}^{\pm }$ is not specified. The measurement of $p_{3}(\phi_{i}^{\pm }|\phi_{i-1}^{\pm },q_{i-1}^{\pm },Q^{\mp })$ is like that for $p_{2}(\phi_{i}^{\pm }|\phi_{i-1}^{\pm },q_{i-1}^{\pm })$ considered in Fig. 8 with an additional specification on the value for *Q*^∓^, which is achieved by controlling the pulse selector circuit with DDG2 rather than DDG1. The measurement of $p_{1}(q_{i}^{\pm }|\Delta \phi_{i,i-1}^{\pm })$ is like that for *p*_1_(*q_i_*|Δ*t_j_*_−1_) in the constant excitation case with the exception that the Δ*t* control logic is triggered by a particular pulse selected from the pulse selector circuit so that *i* has a specified value, i.e., it is not arbitrary as in the constant excitation case. For the measurements of the conditional distributions $p_{2}(q_{i}^{\pm }|\phi_{i}^{\pm },q_{i-1}^{\pm })$ and $p_{3}(q_{i}^{\pm }|\phi_{i}^{\pm },\phi_{i-1}^{\pm },q_{i-1}^{\pm })$, the values of $\phi_{i-1}^{\pm }$ and $q_{i-1}^{\pm }$ are restricted by the windows defined respectively by DDG4 and SCAl as for the measurement of $p_{2}(\phi_{i}^{\pm }|\phi_{i-1}^{\pm },q_{i-1}^{\pm })$ shown in Fig. 8.

It is obvious that the system in Fig. 4 can be con- figured to measure other types of conditional distributions such as $p_{4}(q_{i}^{\pm }|\phi_{i}^{\pm },\phi_{i-1}^{\pm },q_{i-1}^{\pm },Q^{\mp })$. However, the operation of the system has only been tested for distributions like those listed in Tables 4 and 5. The cycle selector allows triggering of DDG1 and DDG4 only after *n* cycles have occurred. By using this feature it is possible to check for memory propagation from half-cycles that occurred prior to the most recent half-cycle such as would be indicated by the conditional distributions $p_{1}(\phi_{ij}^{\pm }|Q_{k}^{\mp })$ with *k > j.*

## 4. Measurement System Components

This section provides detailed information about the individual circuits that were designed for use with the measurement configurations shown in Figs. 3 and 4. Some of the circuits, such as the pulse-sorter circuit in Fig. 3 used for measurement of *p*_0_(Δ*t_j_*) have previously been described [24] and remain unchanged. These circuits are not covered in this section. Some such as the Δ*t* control logic circuits (parts A and B) have been revised and are included here. Circuits that have features specific to the measurement of phase-correlated distributions such as the gated integrator, pulse selector, and gated single-channel analyzer in Fig. 4 have not previously been described and are covered here. Some circuits such as the zero-crossing detector, pulse counter-cycle selector, and absolute-value selector are considered to have well known design and operating characteristics [31] and are not included here. Other circuits not considered here are those that are commercially available such as the digital-delay generators, time-to-amplitude converters, linear pulse amplifiers, and multichannel analyzer. The description of each circuit given below includes both circuit and associated pulse diagrams.

### 4.1 Time-Interval Control Logic (Parts A and B)

The Δ*t* control logic circuits described in this section have replaced the circuits previously shown in Figs. 2 and 4 of Ref. [24]. The designs have been improved to extend the measurement capabilities of the system and to eliminate or reduce errors previously noted [1].

#### 4.1.1 Part A

**Fig. 9:**
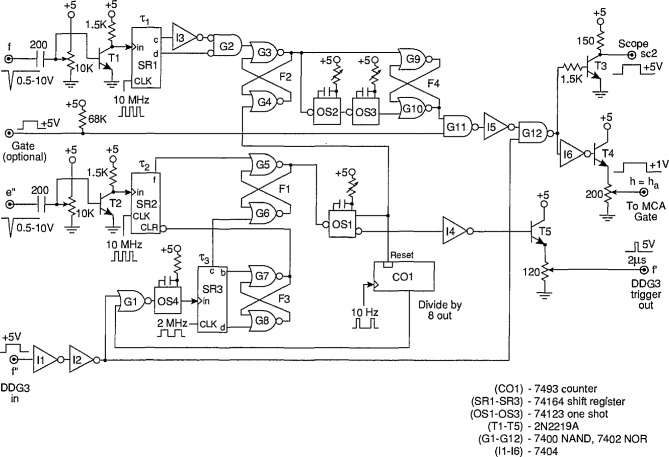
Diagram of the time interval (Δ*t*) control-logic circuit, part A. The individual integrated circuit components are specified in the legend.

**Fig. 10:**
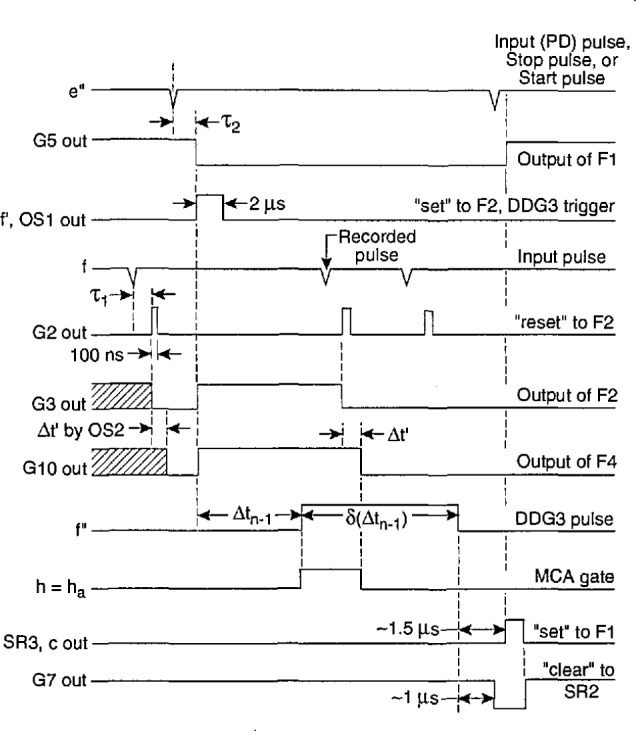
Pulse timing diagram for the different indicated circuit locations in the Δ*t* control logic circuit (part A) shown in Fig. 9.

The function of the Δ*t* control logic (part A) is to control a digital-delay generator (DDG3 in Figs. 3 and 4) to enable either the gate to the MCA (G2 in Figs. 3 and 4) or the gate for the stop pulse in the pulse-selector circuit of Fig. 9. The operation of the circuit can be understood with the aid of the pulse diagram shown in Fig. 10.

Unlike the circuit previously described in Ref. [24], the buffer amplifiers defined by the transistors T1 and T2 are connected to separate inputs (f and e″). For some applications, such as the measurement of *p*_1_(*q_j_*|Δ*t_j_*_−1_), the inputs f and e″ are connected together, and in other applications, such as the measurement of *p*_2_(*q_j_*|*q_j_*_−1_, Δ*t_j_*_−1_) these inputs are connected to different locations, e.g., by switch S2 and Fig. 3. The gain of these buffers is adjusted so that their output is a 5 V logic pulse independent of the peak voltage of the input pulse. Ideally the input pulse voltage should lie within the range of 0.3 to 10 V corresponding to the range accepted by the MCA. For the systems shown in Figs. 3 and 4, the range of input pulse voltages is usually determined by the gain setting of the input amplifier Al.

The outputs of the buffer amplifiers (T1 and T2 in Fig. 9) trigger the 10-MHz serial shift registers SRI and SR2 that serve the purpose of delaying the pulses by the times *τ*_1_ and *τ*_2_ respectively. A pulse appearing at e″ is allowed to trigger the digital-delay generator (DDG3) at output f if the flip-flop circuit F1 defined by gates G5 and G6 is in the proper “initial-condition” state. If it is in this state, then the output of SR2 will cause it to change state after a delay of *τ*_2_ = 600 ns, and the output of G5 will drop to zero thus triggering the one shot OS1. The one-shot produces a 2 *μ*s pulse for triggering DDG3 and also sets flip-flop F2 which indirectly results in the enabling of gate G12. If G12 is enabled, the returning pulse from DDG3 which appears at the f input will be allowed to pass to the output h that is used to control the gate to the MCA or the gate of the pulse selector.

A pulse appearing at the f input will disable G12 and prevent transfer of the DDG3 output pulse to h. In Fig. 10, the DDG3 pulse is indicated to have a delay Δ*t_n_*_−1_ and a width *δ*(Δ*t_n_*_−1_). The circuit is thus designed not to record a pulse in the MCA if that pulse follows the pulse which triggered DDG3 within a time less than Δ*t_n_*_−1_*+ τ*_2_. This feature ensures proper measurement of distributions conditioned on a fixed time separation, e.g., *p*_1_(*q_j_*|Δ*t_j_*_−1_), where it is required that Δ*t_j_*_−1_ fall within the range Δ*t_j_*_−1_ to Δ*t_j_*_−1_ *+ δ*(Δ*t_j_*_−1_) defined by DDG3.

If a pulse appears at f, it will cause F2 to be reset after a delay of *τ*_1_ = 300 ns set by SRI. The delay *τ*_1_ must be less than *τ*_2_ in order to prevent immediate resetting of F2 for coincident inputs at f and e″. The pulse, generated by OS3, that initiates the disabling of gate G12 is delayed by a short interval defined by one-shot OS2. This small added delay (Δ*t*′ in Fig. 10) is necessary to ensure that the MCA gate stays enabled long enough to allow recording of any pulse that appears within the desired time interval.

The circuit is reset by the pulse returned to input f by DDG3. The one-shot OS4 is triggered by the trailing edge of the DDG3 pulse independent of whether or not the gate G12 was disabled before time Δ*t_n_*_−1_ *+ δ*(Δ*t_n_*_−1_) by an intermediate or recorded input pulse. The output of OS4 is delayed by the shift register SR3. Two of the outputs from SR3 control flip-flop F3 (gates G7 and G8) which in turn clears the contents of SR2 to prevent simultaneous setting and resetting of Fl. Another out- put of SR3 then sets Fl to the initial condition which allows DDG3 to be triggered at output f by the next pulse to appear at e″. Generally, any pulse that appears at e″ will also appear at f so that F2 will also be reset in the event that it was not already reset.

The circuit has an additional, optional “gate” input which allows the output pulse at h to be gated by an independent source. This gate is also designated by G3 in Fig. 4 and used for the measurement of $p_{2}(q_{i}^{\pm }|\phi_{i}^{\pm },Q^{\mp })$. The present Δ*t* control logic circuit has an auto-reset capability similar to that described previously [24] so that a reset pulse will appear at G1 to initialize Fl within a time of 0.8 s if for some reason a pulse is not returned from DDG3. If it is necessary to record times longer than 0.8 s, then the 10 Hz auto-reset clock can be replaced with one of lower frequency.

#### 4.1.2 Part B

**Fig. 11:**
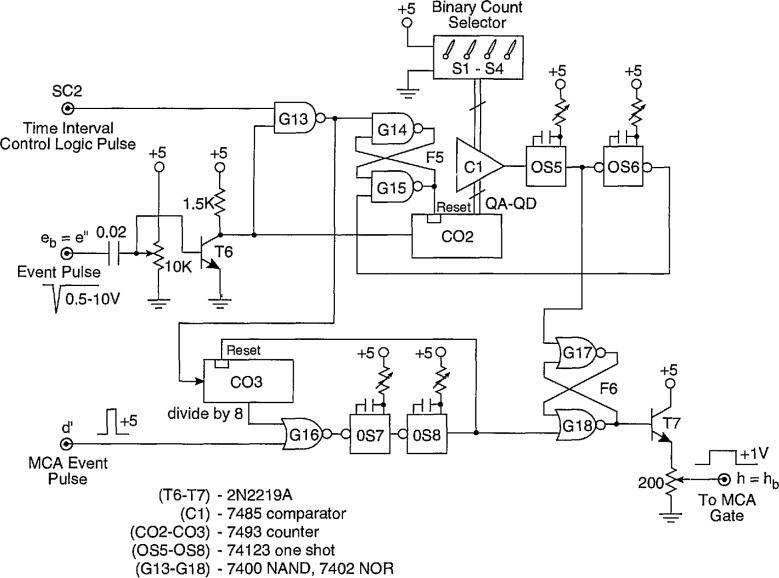
Diagram of the Δ*t* control logic circuit, part B. The individual integrated-circuit components are specified in the legend.

Part B of the Δ*t* control logic is required together with part A when measurements of the conditional pulse-amplitude distributions *p*_1_(*q_j_*|Δ*t_j_*_−i_) are made for *i* > 1 as discussed in Sec. 3.1. The version of the circuit shown in Fig. 11 differs from that described in our earlier work (Fig. 4 of Ref. [24]) which only enabled measurement of *p*_1_(*q_j_*|Δ*t_j_*_−2_) corresponding to the case where *i = 2.* The present circuit incorporates a pulse counting feature that allows determination of distributions for *i* > 2.

**Fig. 12:**
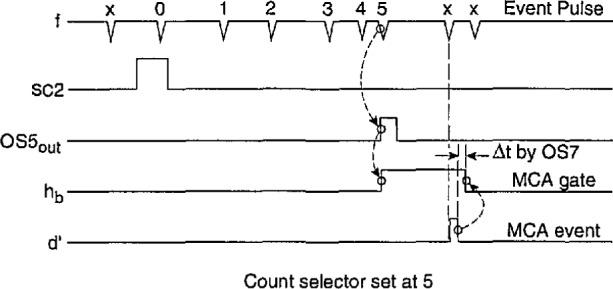
Pulse timing diagram for the Δ*t* control logic (part B) shown in Fig. 11. It is assumed in this example that the binary count selector is set by switches S1–S4 to the value 5.

The operation of the circuit shown in Fig. 11 can be understood from a consideration of the pulse diagram shown in Fig. 12. The pulse time separation between the (*j* − *i +* 1) and (*j* − i)th events (see Fig. 1) is restricted by the delay and pulse width settings of DDG3. The output of DDG3 is controlled by part A as described in the previous section so as to ensure that Δ*t_j_*_−i_ is properly defined as a time separation between adjacent pulses. The operation of the Δ*t* control logic (part B) is thus initiated by the simultaneous occurrence of an event (PD) pulse at input e_b_ = e″ and a pulse from the h = h_a_ (or equivalently SC2) output of the Δ*t* control logic (part A) at input SC2. An event occurring in coincidence with the SC2 input sets flip-flop F5 (gates G14 and G15) which in turn allows the counter CO2 to count subsequent event pulses. When the output of the counter corresponds to a binary number equal to *i* − 2, as determined by the settings of switches S1–S4, the comparator C1 triggers the one-shot OSS. The output of OS5 sets flip-flop F6 (gates G17 and G18) which enables the gate (G2 in Fig. 3) of the MCA at output h = h_b_. It also triggers the one-shot OS6 which in turn resets F5 and consequently also the counter CO2. The MCA will record the next *(j* th) event to occur after G3 is opened independent of its time separation from the preceding (*j* − 1)th event. Immediately after this event is recorded, the MCA sends a 5 V “event pulse” to the input d′. After a short delay determined by the one-shots OS7 and OS8, the event pulse resets F6 and thereby disables the MCA gate. The circuit is then ready to be triggered by the next coincidence to occur at inputs SC2 and e_b_ = e″. If for any reason the MCA fails to return an event pulse, e.g., the *j* th pulse was too small to be recorded, then the counter CO3 automatically provides a reset for F6 after 8 successive coincident pulses have appeared at SC2 and e_b_ = e″. 4.2 Pulse Selector

### 4.2 Pulse Selector

**Fig. 13:**
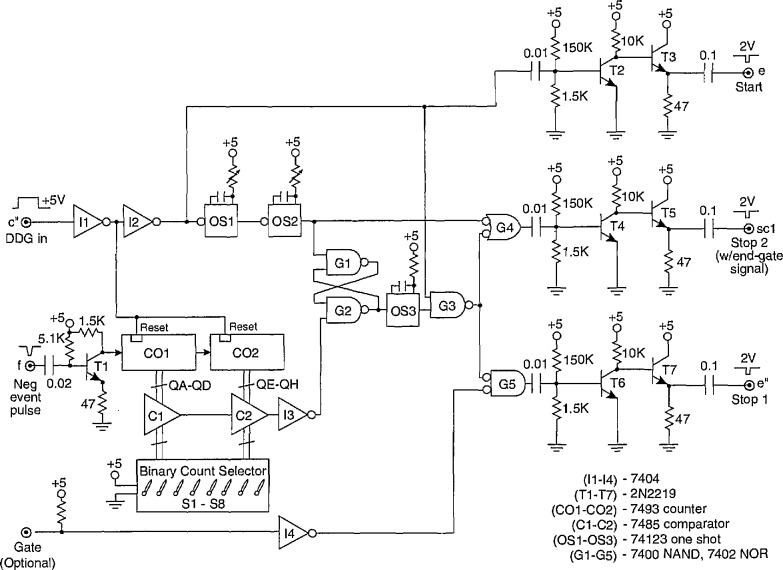
Diagram of the pulse-selector circuit used for the measurement system shown in Fig. 4. The individual circuit components are specified in the legend.

**Fig. 14:**
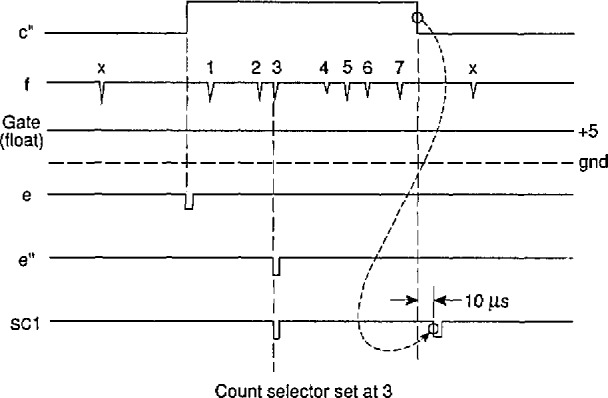
Pulse timing diagram for the pulse-selector circuit shown in Fig. 13. It is assumed here that the binary count selector is set to select the third pulse to occur within the time interval defined by the duration of the pulse appearing at input c″.

A diagram of the pulse-selector circuit is shown in Fig. 13. The associated pulse diagram is shown in Fig. 14. This circuit is used to select a particular numbered event that occurs after a specified time or phase. It is used in the system shown in Fig. 4 to measure the conditional phase or amplitude distributions of specific pulses that occur within well-defined phase windows of the excitation voltage, e.g., $p_{1}(\phi_{i}^{\pm }|Q^{\mp })$, *i* = 1,2,3…

The pulse selector produces a − 2 V “start” pulse at output e that is coincident with the rising edge off a + 5 V digital-delay generator pulse applied to input c″. This DDG pulse is assumed to define a fixed phase or time window, and therefore the output at e occurs at a known phase or time, i.e., it provides the appropriate phase reference point. The circuit is designed to produce − 2 V “stop” pulses at outputs e″ (Stop 1) and SC1 (Stop 2) that are coincident with the *i* th pulse to occur after the phase-reference point, i.e., after the “start” pulse. The value of *i* is determined by the setting of the binary count selector (switches S1 – S8).

In the operation of this circuit, the pulse counters CO1 and CO2 are enabled by the DDG window. The negative event pulses that appear at input f are then counted by CO1 and CO2. The binary outputs of these counters are sensed by the comparators C1 and C2. If and when the binary number presented by the counter outputs equals the 8-bit binary number selected by the terminal switches, i.e., for *i* = 1 to 256 pulses, the output of C2 goes from 0 to +5V and sets the flip-flop defined by gates G1 and G2. This flip-flop triggers the one-shot OS3 that produces the “stop” pulses that ultimately appear at e″ and SC1.

At the falling edge of the DDG pulse, the counters CO1 and CO2 are reset and the one-shots OS1 and OS2 are triggered. The output of OS2 resets the flip-flop and delivers a “stop” pulse to SC1 in coincidence with the end of the DDG window. This pulse does not appear at the e″ output. The “end-of-window” stop pulse provides another phase mark that may be useful in some applications.

The appearance of the stop pulse at e″ can also be controlled by another external pulse applied to the “gate” input. This option is used for the system shown in Fig. 4 for the measurement of various conditional phase distributions as indicated in Table 5.

### 4.3 Single-Channel Analyzer

**Fig. 15:**
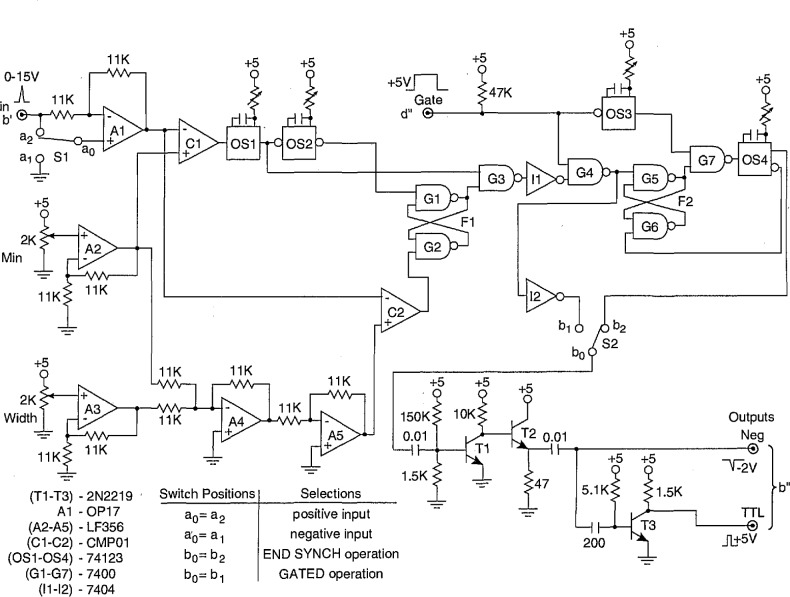
Circuit diagram for the gated single-channel analyzer. The individual integrated-circuit components and switch configurations of the different modes of operation are shown in the legend.

**Fig. 16:**
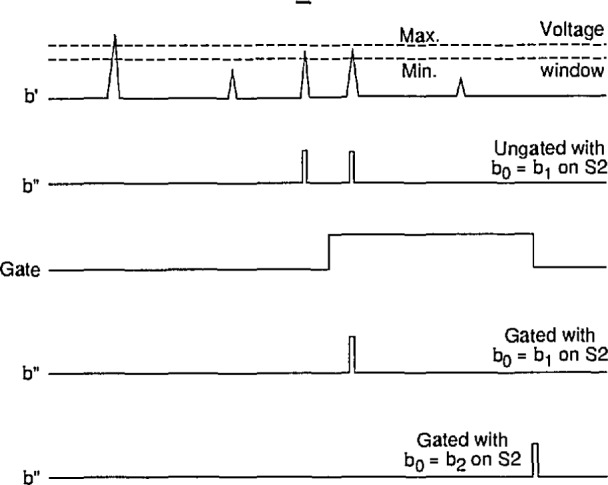
Pulse timing diagram corresponding to the different possible operating modes for the single-channel analyzer shown in Fig. 15.

**Fig. 17:**
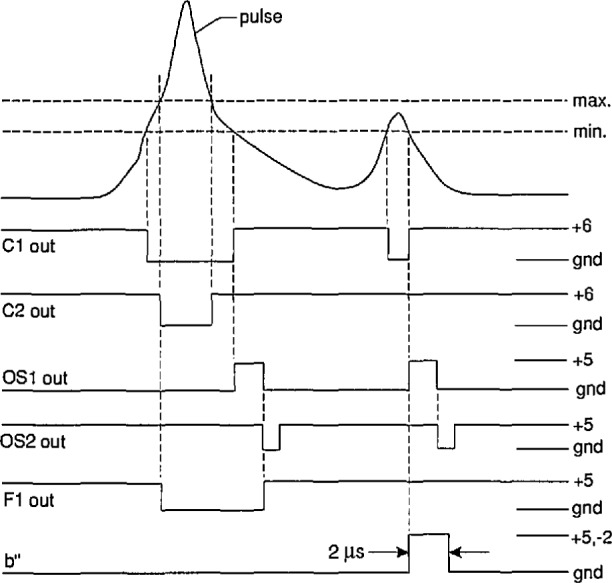
Pulse timing diagram for the indicated circuit locations of the single-channel analyzer shown in Fig. 15.

The single-channel analyzer circuit designed for the measurement systems shown in Figs. 3 and 4 is presented in Fig. 15. The operation of this circuit is indicated by the pulse diagrams shown in Figs. 16 and 17. If the amplitude of the input pulse at b′ lies within a selectable voltage window, then the SCA delivers both a −2 V and +5 V pulse to the indicated output points at b″.

The circuit is capable of either gated or ungated operation. For ungated operation, output pulses are generated at b″ for any input pulse that has an amplitude within the selected window independent of its time of occurrence. For gated operation, output pulses are generated only if the input pulse occurs within a time interval defined by the width of a 5 V gate pulse applied to input d″. Depending on the setting of the switch S2, the output pulses for the gated operation will either appear at a time approximately coincident with the input pulse (with a slight delay) or at a time coincident with the end of the gate pulse. The three possible modes of operation are identified in Fig. 16.

In the operation of this circuit, the event pulse is sensed by amplifier Al, the output of which either follows or inverts the signal depending on the position of switch SI. The output of this amplifier then proceeds to the analog comparators C1 and C2. The other inputs to the comparators are derived from that part of the circuit (amplifiers A2–A5) that define the minimum voltage and width of the window. Voltages in the range of 0 to + 5 V are selected by the two 2 kΩ resistors denoted by “min” and “width” in Fig. 15. These voltages are doubled by amplifiers A2 and A3. Amplifier A4 sums and inverts the outputs of A2 and A3, and amplifier A5 in turn inverts the output of A4. Consequently, the positive input of C1 is a voltage between 0 and +10 V corresponding to twice the “min” value and the positive input of C2 is a voltage between 0 and +15 V equal to twice the value of the “min” plus “width” voltages.

As indicated in Fig. 17, the outputs of C1 and C2 are normally high (+ 6 V) and go negative when the negative input exceeds the positive input. The circuit is designed so that input event pulses with amplitudes below the “min” value are ignored while those exceeding the maximum Value (“min” plus “window”) are inhibited. In the latter case, the inhibition results from the setting of flip-flop F1 (gates G1 and G2) by the output of C2 which in turn disables G3. If the input event pulses fall within the window, then the output of the one-shot OS1 triggered by C1 passes through the gate G3 and becomes the source for the 2 *μ*s output pulse at b″. The output of OS1 also triggers OS2 which resets F1 after a delay sufficient to prevent passage of any pulses through G3 that exceed the maximum value as illustrated in Fig. 17.

For normal gated operation, the switch S2 is set so that b_0_ = b_1_. This allows G4 to be enabled by a + 5 V gate pulse at d″ and thereby permits passage of the pulse from G3 to the output buffer amplifiers (transistors T1–T3). If it is desired to have the output pulses appear at a fixed time corresponding to the end of the gate pulse, then b_0_=b_2_ at S2. In this mode, the output of OS1 passes through G3 and G4 and ultimately triggers flip-flop F2 (gates G5 and G6) which in turn enables G7. The one-shot OS3 is triggered by the falling edge of the gate pulse and thus produces a pulse that passes through G7 and triggers OS4. The output of OS4 resets F2 and also becomes the source of the output pulses at b″.

### 4.4 Gated Integrator

**Fig. 18:**
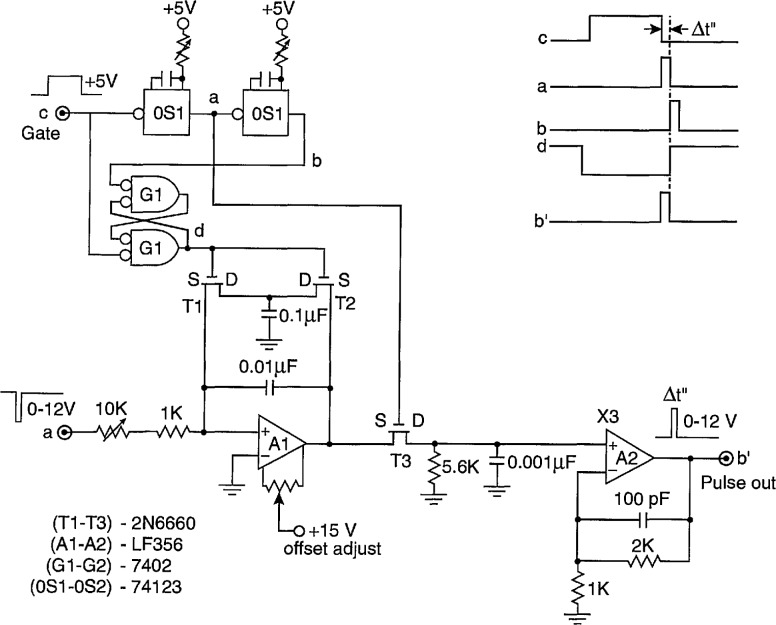
Circuit diagram for the gated integrator.

**Fig. 19:**
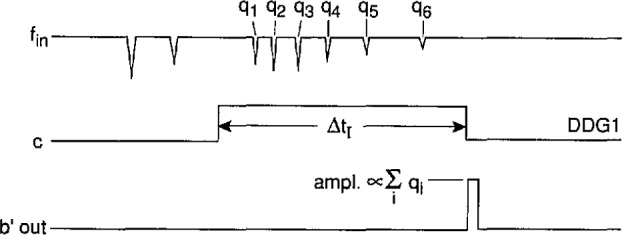
Pulse timing diagram for the gated integrator circuit shown in Fig. 18.

The integrator circuit used for the measurement system in Fig. 4 is shown in Fig. 18 and the corresponding pulse diagram is shown in Fig. 19. The output of the integrator is a pulse with an amplitude in the range of 0 to 12 V directly proportional to the sum of the areas under all pulses that occur within the gate time interval denoted by Δ*t*_I_ in Fig. 19. If all pulses have the same shape so that their amplitudes are proportional to their areas, then the height of the integrator output pulse is also proportional to the sum of the amplitudes of all pulses occurring within Δ*t*_I_.

For the circuit in Fig. 18, the input pulses to the integrating amplifier Al are assumed to be of constant width (~1 *μ*s) with amplitudes in the range of 0 to −12 V. In the absence of a 5 V gate pulse at input c, the flip-flop defined by gates G1 and G2 keeps the field-effect transistors (FET’s) T1 and T2 turned on so that the 0.01 *μ*F integrating capacitor is effectively shorted. The application of a pulse at c changes the state of the flip-flop which then turns off T1 and T2 thus allowing charge to accumulate on the integrating capacitor. At the end of the gate pulse, the one-shot OS1 is triggered and its output momentarily turns on T3 which allows transfer of the integrator amplifier output voltage to amplifier A2 and thereby to the output terminal b′. The trailing edge of the OS1 output pulse also triggers OS2 that generates a pulse to reset the flip-flop which in turn turns on T1 and T2 thereby discharging the integrating capacitor.

## 5. Examples of Results

The purpose of this section is to show examples of data on conditional and unconditional distributions that have been obtained using the systems shown in Figs. 3 and 4 respectively for measurement of dc and ac excited pulsating PD. A detailed discussion of the physical bases for the observed stochastic properties of PD phenomena goes beyond the scope of this paper and can be found in other works [1–4].

### 5.1 Continuous Excitation Process (de-Generated PD)

**Fig. 20:**
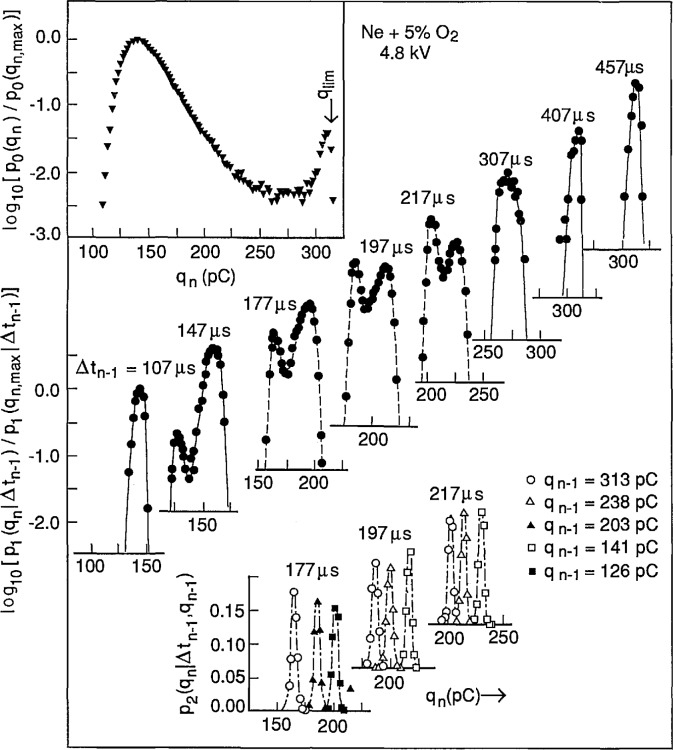
Measured unconditional and conditional pulse-amplitude distributions *p*_0_(*q_n_*), *p*_1_(*q_n_*|Δ*t_n_*_−1_), and *p*_2_(*q_n_*|*q_n_*_−1_, Δ*t_n_*_−1_) at the indicated “fixed” values for Δ*t_n_*_−1_ and *q_n_*_−1_ for negative-corona discharge pulses generated using a point-plane electrode gap in a Ne + 5% O_2_ gas mixture (see Ref. [1]).

Shown in Fig. 20 are examples of the measured unconditional and conditional pulse-amplitude distributions *p*_0_(*q_n_*), *p*_1_(*q_n_*|Δ*t_n_*_−1_) and *p*_2_(*q_n_*|Δ*t_n_*_−1_,*q_n_*_−1_) for negative corona (Trichel) pulse discharges generated with a point-plane electrode gap in a neon-oxygen gas mixture at atmospheric pressure (100 kPa). The unconditional and first-order conditional distributions are plotted on a logarithmic scale and normalized to the maximum values to facilitate comparisons of the various distributions. The pulse amplitudes are expressed in units of (pC) as explained in previous work [1,27] (also see Sect. 6.1). The dependence of the first-order distributions *p*_1_(*q_n_*|Δ*t_n_*_−1_) on Δ*t_n_*_−1_ implies a strong positive dependence of *q_n_* on Δ*t_n_*_−1_, i.e., (Δ*t_n_*_−1_ ↑ ⇒ *q_n_* ↑). This behavior can be explained in terms of the expected influence of the moving negative-ion space-charge cloud from the previous pulse on the electric field in the gap and consequently also on the growth of the next discharge pulse [1].

The dependence of the distributions *p*_2_(*q_n_*|Δ*t_n_*_−1_, *q_n_*_−1_) on *q_n_*_−1_ for a fixed Δ*t_n_*_−1_ implies a negative dependence of *q_n_* on *q_n_*_−1_, i.e., (*q_n_*_−1_ ↑ ⇒ *q_n_* ↓, Δ*t_n_*_−1_). This behavior can be explained from consideration of the *size* of the space-charge cloud from the previous event on the growth of a discharge pulse. The data for both the first and second order pulse-amplitude distributions clearly demonstrate the importance of memory effects in determining the stochastic behavior of this discharge phenomenon.

**Fig. 21:**
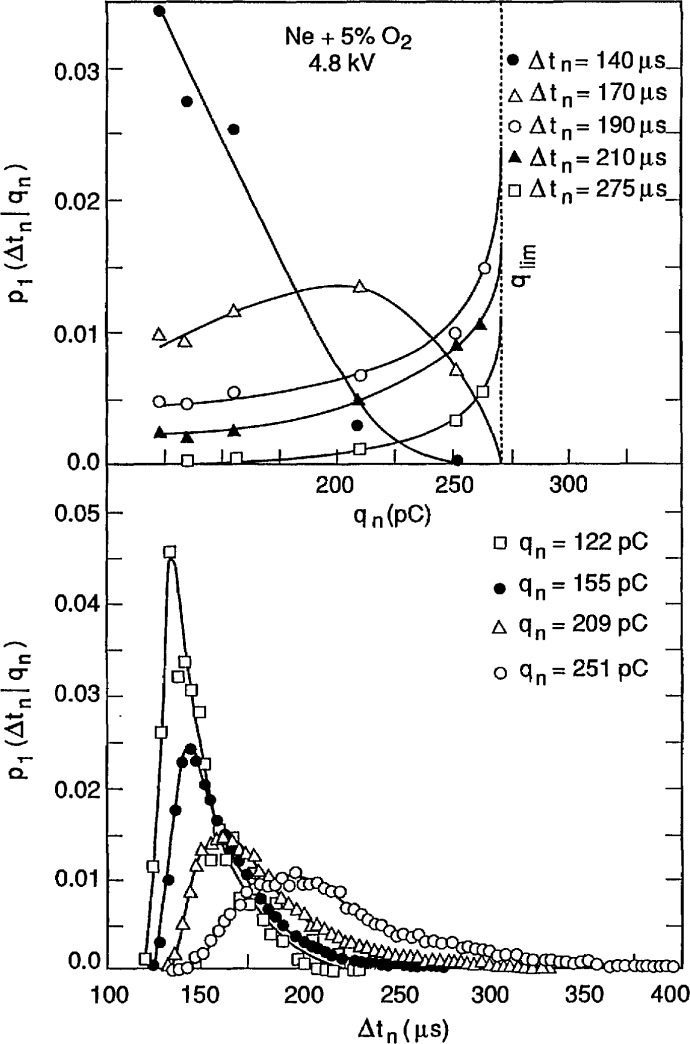
Measured conditional time-separation distributions *p*_1_(Δ*t_n_|q_n_*) at the indicated values for Δ*t_n_* or *q_n_* for negative-corona discharge pulses generated using a point-plane electrode gap in a Ne + 5% O_2_ gas mixture under conditions similar to those that yielded the data shown in Fig. 20 (see Ref. [1]).

The dashed lines shown for the first-order distributions at Δ*t_n_*_−1_ = 177, 197, and 217 μs were calculated using the integral expression [1]

$$\begin{array}{l} p_{1}(q_{n}|\Delta t_{n-1})=p_{0}{\left(\Delta t_{n-1}\right)}^{-1}\int_{0}^{\infty }p_{0}(q_{n-1})\\ p_{1}(\Delta t_{n-1}|q_{n-1})p_{2}(q_{n}|q_{n-1},\Delta t_{n-1})dq_{n-1} \end{array}$$ (13)

with numerical data obtained for the distributions shown in the right-hand side. Data for the conditional time-interval distribution, *p*_1_(Δ*t_n_|q_n_*), used in the integral are shown in Fig. 21. It is interesting to note that in this case 〈Δ*t_n_*(*q_n_*)〉 increases as *q_n_* increases, i.e., (*q_n_* ↑ ⇒ Δ*t_n_*_−1_ ↑). This means that the larger the previous event, the longer on average will be the time spacing between this event and the next event. This has been explained in terms of the influence of the electric field generated by space charge from earlier discharge pulses in suppressing the release of electrons from the cathode needed to initiate subsequent pulses [1].

It is evident from the results shown here that it would be impossible to find a physical interpretation of measured unconditional pulse-amplitude distributions without information about the memory effects revealed by the conditional distributions. The unconditional amplitude distribution is related to the time-interval distribution *p*_0_(Δ*t_n_*) and the conditional distribution *p*_1_(*q_n_*|Δ*t_n_*_−1_) through Eq. (7). The first-order conditional distribution is in turn related to higher order distributions through Eq. (13). An unraveling of memory effects is a required step toward understanding the observed stochastic properties of random point processes such as reported here for the Trichel-pulse discharges.

### 5.2 Periodic Time Varying Excitation Process (ac-generated PD)

**Fig. 22:**
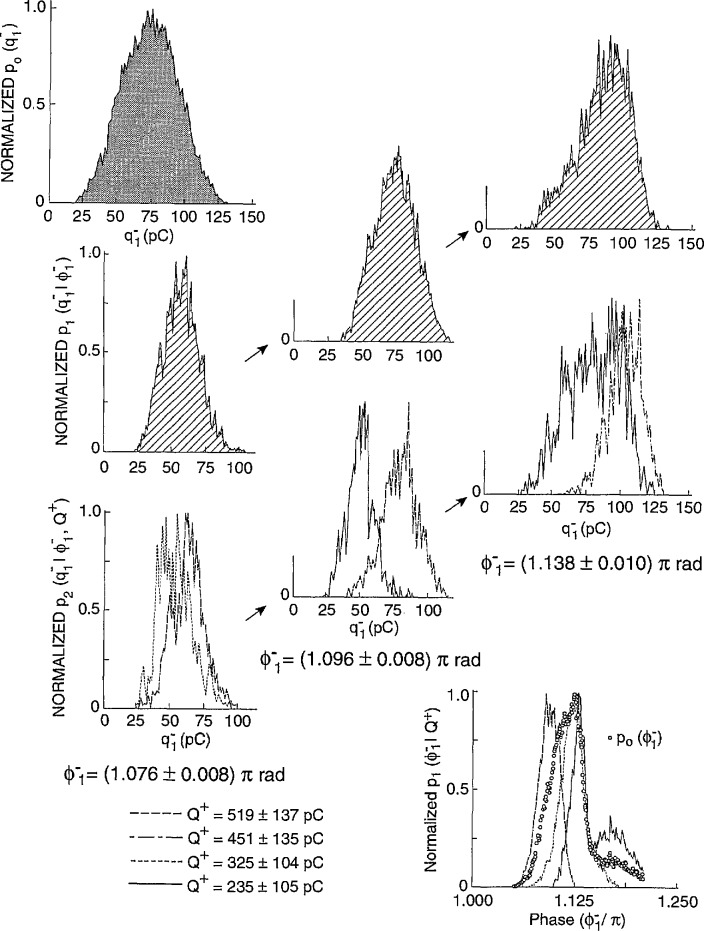
Measured conditional and unconditional amplitude distributions of the first negative PD pulse at the indicated values of $\phi_{1}^{-}$ and *Q*^+^ for a point-to-dielectric discharge gap spacing of 1.2 mm and an applied alternating voltage of 3.0 kV rms at a frequency of 200 Hz. Also shown are the conditional and unconditional phase-of-occurrence distributions for the same pulse. All distributions have been arbitrarily normalized to the maximum values.

Data were obtained in this case for partial dis- charges generated by applying a sinusoidal alternating voltage to a point-dielectric discharge gap in air. Preliminary results from these measurements have recently been reported [2, 4, 32]. Figure 22 shows examples of measured unconditional and conditional pulse-amplitude distributions of the first negative pulse to appear in each cycle. Also shown are the unconditional and conditional phase-of-occurrence distributions for this pulse. These results were acquired after observing the discharge pulses for many thousands of cycles of the applied voltage.

The data shown in Fig. 22 were obtained using a stainless-steel point electrode positioned over a large, flat polytetrafluoroethylene (PTFE) dielectric surface in room air at a temperature of 23 °C. The tip of the stainless-steel electrode had a radius-of-curvature of 0.05 mm and was separated from the PTFE surface by a gap of 1.2 mm. A 200 Hz, 3.0 kV rms sinusoidal voltage was applied to the gap.

All distributions shown in Fig. 22 have been arbitrarily normalized to the maximum values. The indicated values for *Q*^+^ correspond to the integrated charge associated with all positive PD events in the previous half-cycle [see Eq. (4)] and define the type of line used to represent the data. In the case of the second-order distributions, $p_{2}(q_{1}^{-}|\phi_{1}^{-},Q^{+})$, the fixed phase windows are defined directly under the data to which they apply.

There are clear indications from these data of the significance of memory propagation in deter- mining the stochastic behavior of the phenomenon. The data for $p_{1}(\phi_{1}^{-}|Q^{+})$ indicate that the larger the value *of Q*^+^, the smaller is the value of the mean phase-of-occurrence of the first negative PD pulse. This means that $\phi_{1}^{-}$ has a negative dependence on *Q*^+^, i.e., $\left(Q^{+}↑\Rightarrow \phi_{1}^{-}↓\right)$. The data for $p_{2}(q_{1}^{-}|\phi_{1}^{-},Q^{+})$ show that $q_{1}^{-}$ is positively dependent upon *Q*^+^ for a fixed phase-of-occurrence $\phi_{1}^{-}$, i.e., $\left(Q^{+}↑\Rightarrow q_{1}^{-}↑,\phi_{1}^{-}\right)$. The data for $p_{1}(q_{1}^{-}|\phi_{1}^{-})$ show that the mean value of the first negative pulse amplitude increases with its phase-of-occurrence. This distribution is related to the unconditional distribution *p*_0_(*Q*^+^) and the other conditional distributions shown in Fig. 22 by the expression

$$\begin{array}{l} \;p_{0}(\phi_{1}^{-})p_{1}(q_{1}^{-}|\phi_{1}^{-})=\int_{0}^{\infty }p_{0}(Q^{+})\\ p_{1}(\phi_{1}^{-}|Q^{+})p_{2}(q_{1}^{-}|\phi_{1}^{-},Q^{+})dQ^{+}. \end{array}$$ (14)

The corresponding data for *p*_0_(*Q*^+^) are not shown. At present, it has not been possible to obtain enough data on the required distributions under stationary discharge conditions to verify that Eq. (14) is indeed consistent with the experimental results.

It has recently been shown [33] that the types of stochastic behavior for ac-generated PD reported here are consistent with theoretical predictions derived from a Monte-Carlo simulation of the phenomenon. The primary long-term (cycle-to-cycle) mechanism for memory propagation is that due to electric charge accumulation on the dielectric surface during a PD event. It is well known that a quasi-permanent surface-charge distribution can exist on a solid insulating surface for times that are long compared to typical periods of the excitation voltage [34H37]. A significant fraction of the charge deposited on a dielectric surface by a PD event will remain to affect the local electric-field strength at the site where the next PD event is initiated. Both the probability for PD initiation and the distribution of the PD amplitudes depends at any given time on the local instantaneous electric-field strength. As in the case of dc-generated Trichel pulses, short-term pulse-to-pulse memory propagation can also exist for ac-generated PD. Mechanisms for memory propagation in this case could include moving ion space charge [1, 38], diffusion of metastable excited species [39], or a rapid redistribution of charge on a dielectric surface following a PD event [2, 40].

## 6. Calibrations and Sources of Error

In the discussion about the earlier version of the stochastic analyzer [24], several sources of systematic error were considered. These were primarily errors associated with the finite digital-delay generator time window and the finite reset time of the time-to-amplitude converter. These among other sources of error need to be considered in making interpretations of the measured distributions and in judging the validities of consistency analyses performed using relationships like Eqs. (7), (8), and (13). It is, for example, important in considering the use of Eq. (7) in checking consistency among the measured distributions *p*_0_(*q_j_*), *p*_0_(Δ*t_j_*), and *p*_1_(*q_j_*|Δ*t_j_*_−1_) to know the extent to which *p*_1_(*q_j_*|Δ*t_j_*_−1_) represents the true conditional distribution for a fixed Δ*t_j_*_−1_ [see Eq. (9)]. It is also important that measurements of Δ*t_j_* using a TAG and the determination of Δ*t_j_*_−1_ using the combined DDG and Δ*t* control logic yield identical time separations. Any error in one of these circuits relative to the other can cause difficulties in performing the integration implied by Eq. (7). Thus, for example, it is generally necessary to make corrections for the delay τ_2_ introduced by the Δ*t* control logic circuit (see Fig. 10). In this section we consider the possible sources of error in the measurement of various amplitude, phase-of-occurrence, time-separation, and integrated pulse (charge) distributions that can be measured with the system described above. Methods for calibration and testing of system performance are also discussed.

### 6.1 Amplitude Distributions

#### 6.6.1 Pulse Shape Considerations

The method for calibration of pulse amplitudes for PD has been described previously [27]. One could, in the simplest case, directly apply pulses of a known amplitude to the input of the system (amplifier Al of Figs. 3 and 4) and then record the MCA channel numbers corresponding to pulses of different amplitude. In most cases, however, it is desirable that the simulated input pulses used for calibration be similar in shape to those observed for the phenomenon of interest. This is especially required in the case of partial-discharge measurements where the amplitude of the recorded PD event is supposed to be proportional to the discharge intensity. It has been shown [1, 27] that, for the types of PD phenomena considered in the previous section, the recorded pulse amplitude is proportional to the net charge generated during the pulse provided the width of the impulse response for the detection system is very large compared to the intrinsic width of a typical discharge pulse. Under this condition, the shape of the recorded pulse is governed primarily by the impulse response of the detection circuit. The width of the impulse response for the detection system used to obtain the data in Figs. 20–22 is approximately 1.5 μs compared to a typical intrinsic PD pulse width of 1 to 11 ns.

Pulses for some types of PD phenomena such as pulsating corona in air are known to have tails that are long compared to the 1.5 *μ*s impulse response width corresponding to the conditions under which the data in the previous section were taken. In such cases, not only is the measured pulse amplitude no longer directly proportional to the total charge generated by the PD event, but there may also exist the possibility that the system will sample the tail of the pulse one or more times in addition to its peak value. As noted in our earlier work [27], this problem can occur if the system sampling rate, governed primarily by the dead time of the MCA, is sufficiently high. (The MCA dead time is approximately 2 μs for the system used in this work). If this problem occurs, the measured amplitude distribution will be artificially enhanced at the low-amplitude end due to recording of the tails. A similar problem of pulse amplitude distribution distortion is known to occur in cases where the PD occurs as bursts of pulses in which: 1) the spacing between pulses is comparable to or shorter than the detector impulse response time or MCA dead-time, i.e., the system sampling time; 2) the duration of the burst is comparable to or longer than the system sampling time; and 3) there is a high degree of correlation among the amplitudes of pulses within a burst. Such short-duration, burst-type PD pulses for which pulse amplitudes are highly correlated are known to occur under some conditions [4, 27, 41].

The accidental sampling of pulse tails can also be a problem when measuring conditional pulse-amplitude distributions as discussed in our earlier work [24]. In this case, a problem arises if the MCA is gated on by the Δ*t* control logic at precisely the time when an event pulse is decaying, i.e., after a peak has occurred. The problem has been minimized in the present system by a combination of shaping the pulses that enter the MCA and by minimizing the delay time Δ*t*′ indicated in Fig. 10 as determined by the one shots OS2 and OS3 in Fig. 9. These adjustments were sufficient to yield acceptable results for the types of PD phenomena considered in this work. Complete elimination of this problem is difficult, but can be at least partially achieved by modification of the Δ*t* control logic so that it senses if a pulse has occurred within a short time of approximately one pulse width before the DDG3 pulse is returned to input f″ in Fig. 9. If a pulse does occur within that time, then a condition can be set to force a delay in the opening of the MCA gate at output h.

#### 6.1.2 Effect of Amplifier Nonlinearities

**Fig. 23:**
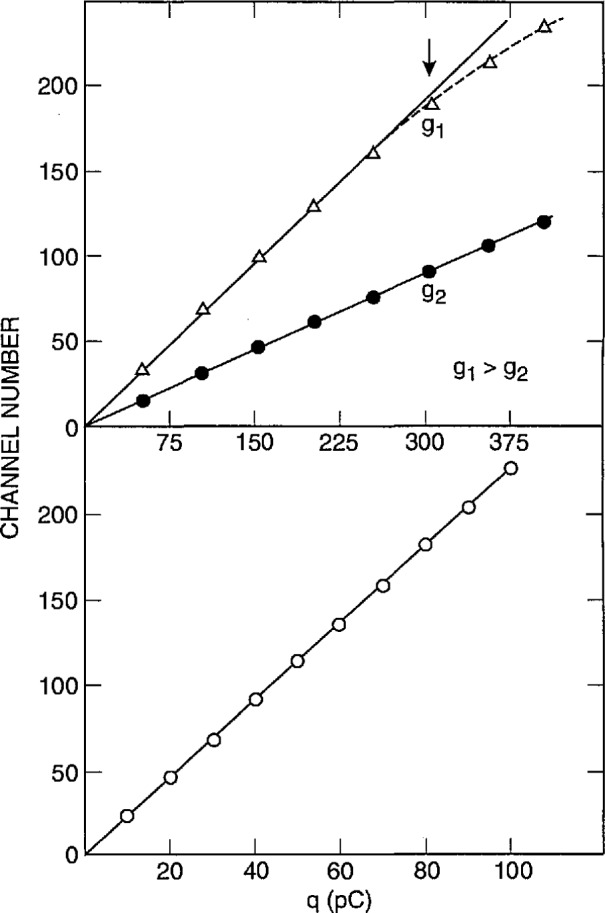
Examples of amplitude-calibration curves for two different ranges of amplitudes and two different amplifier gains, *g*_1_, and *g*_2_, that apply to the measurement of PD pulse-amplitude distributions. The vertical arrow indicates the onset of gain saturation for the highest range and gain *g*_1_.

Another possible source of distortions in measured conditional or unconditional pulse-amplitude distributions is that associated with nonlinearities in the gains of pulse amplifiers used in the detection circuitry or elsewhere in the measurement system, e.g., amplifier Al in Figs. 3 and 4. Figure 23 shows typical examples of calibration curves used in analyzing the data on pulse-amplitude distributions such as those shown in Figs. 20 and 22. Shown are plots of amplitude in charge (pC) versus MCA channel number for two different ranges of amplitude and for two different gain settings (*g*_1_ and *g*_2_) of the amplifier Al for the same input amplitude range. The onset of gain saturation in the preamplifier that detects a pulses is indicated by the vertical arrow pointing to a place on the curve corresponding to the highest gain and highest amplitude range.

It is desirable that the response of the pulse amplifier be as linear as possible. Deviations from linearity introduce complications in determining the true amplitude distribution from the measured data as will be shown below. The data recorded by the MCA can be represented by the array of numbers *N*(*k*) where *k* is the channel number and *N*(*k*) is the number of events stored in *k.* For a 256 channel MCA, *k* is restricted to integer values between 1 and 256. From calibration data such as shown in Fig. 23, one can relate the *N*(*k*) data to the true amplitude distribution by

$$N(k)\Delta k=N_{0}\int_{q(k_{L})}^{q(k_{U})}p_{j}(q)dq$$ (15)

where *N*_0_ is a normalization constant and *p_j_*(*q*) denotes a “true” *j* th order conditional amplitude distribution. If a single channel width is assumed so that Δ*k* = 1, then *q* (*k*_U_) and *q* (*k*_L_) are defined here to correspond respectively to the values $k_{U}=k+\frac{1}{2}$ and $k_{L}=k-\frac{1}{2}$.

If the pulse amplification is linear, we can write

$$k=\alpha_{0}+\alpha_{1}q,$$ (16)

where *α*_0_ and *α*_1_ are constants. *If p_j_*(*q*) is slowly varying over the interval [*q* (*k*_U_), *q* (*k*_L_)] so that

$$\frac{p_{j}(q(k_{U}))-p_{j}(q(k_{L}))}{p_{j}(\bar{q})}\ll 1,$$ (17)

where $\bar{q}\equiv [q(k_{U})+q(k_{L})]/2$, then in the linear case

$$N(k)\approx N_{0}p_{j}(\bar{q})[q(k_{U})-q(k_{L})]$$ (18)

$$=N_{0}\left(\frac{1}{\alpha_{1}}\right)p_{j}(\bar{q}).$$ (19)

Equations (18) and (19) imply that *N*(*k*) versus *k* is a discretized approximation to the true distribution *p_j_*(*q*).

For cases where the response is nonlinear, Eq. (19) is not valid and even Eq. (18) may fail to be a good approximation. If a quadratic dependence is included in Eq. (16) by adding a term *α*_2_*q*^2^ to the right-hand side, then the factor of (1/*α*_1_) in Eq. (19) must be replaced with the factor $\left[1/(\alpha_{1}+2\alpha_{2}\bar{q})\right]$ which depends on $\bar{q}$. For a sufficiently large quadratic contribution, it is necessary to consider this $\bar{q}$ dependent factor in attempts to estimate $p_{j}(\bar{q})$ distribution from the raw *N(k)* versus *k* data.

Under conditions of severe nonlinearity, it may become impossible to extract meaningful information about *p_j_*(*q*) from the MCA data. One such case is that encountered when the input amplifier gain approaches saturation. As an example of this case we consider an amplifier that begins to saturate for *q* ⩾ *q*_s_. The calibration curve for this case is represented mathematically by:

$$k=\alpha_{0}+\alpha_{1}q,\;q<q_{s}$$ (20)

$$k=\alpha_{0}+\alpha_{1}^{\prime }[1-\exp(-\beta q)],\;q>q_{s},$$ (21)

where $\alpha_{1}^{\prime }$ and *β* are constants and the gain is assumed to be linear for *q < q*_s_. In order that both *k* and d*k/*d*q* be continuous at *q* = *q*_s_, the coefficients must satisfy the relationships

$$\alpha_{1}^{\prime }=\frac{\alpha_{1}q_{s}}{\left[1-exp(-\beta q_{s})\right]}$$ (22)

and

$$(\beta q_{s}+1)\exp(-\beta q_{s})=1.$$ (23)

In the region of saturation (*q* ⩾ *q*_s_) we have

$$\frac{dk}{dq}=\alpha_{1}\exp[-\beta (q-q_{s})].$$ (24)

Equation (24) implies that for a fixed increment of channel number, e.g., Δ*k* = 1, the difference between *q* (*k*_U_) and *q* (*k*_L_) increases exponentially with *q*, i.e.,

$$q(k_{U})-q(k_{L})\sim exp[\beta (q-q_{s})].$$ (25)

In this case, Eq. (18) may not hold and, in general, one must resort to Eq. (15) to relate *N*(*k*) to *p_j_*(*q*). The range of *q* values over which Eq. (15) must be integrated can become large enough under some conditions to prevent the determination of reasonable estimates for *p_j_*(*q*) from the MCA data. Generally, the effect of amplifier saturation is to cause *N*(*k*) to become artificially enhanced at large values of *k*. The problems associated with saturation can usually be avoided by making careful adjustments of amplifier gain.

A reasonable estimate of the true amplitude distribution also requires that the amplitude increment, *q* (*k*_U_) − *q* (*k*_L_), associated with the width of a single channel be small compared to the characteristic width of the distribution. This can usually be assured by appropriate adjustment of the amplifier gain and MCA pulse discrimination level that respectively determine the parameters *α*_1_ and *α*_0_ in Eq. (16). There may exist cases, however, where the ability to make a precise determination of conditional or unconditional distributions is severely limited by the inherent resolution of the MCA.

#### 6.1.3 Noise Broadening

Under low signal-level conditions, distortion of the measured amplitude distributions can result from effects of noise. Specific sources of noise are not identified here, but they could simply be those associated with normal amplifier operation. The type of noise considered in the present discussion is often referred to as “white noise.” The noise is assumed to have constant statistical characteristics during the time of a typical measurement, i.e., it is assumed to be stationary.

Not considered in this discussion are erratic or time-dependent noise such as might appear as random or phase correlated pulses generated by pick-up from sources external to the system of interest. In cases where such externally produced impulses cannot be eliminated by adjusting the amplifier or MCA discrimination levels, it is possible that these impulses will introduce severe distortions, especially if they are narrowly distributed in amplitude, phase, or frequency. Elimination of interference from impulse noise sources can be achieved under some conditions by using shielding or digital-filtering techniques [42, 43]. Discussion of these techniques goes beyond the scope of the present work.

**Fig. 24:**
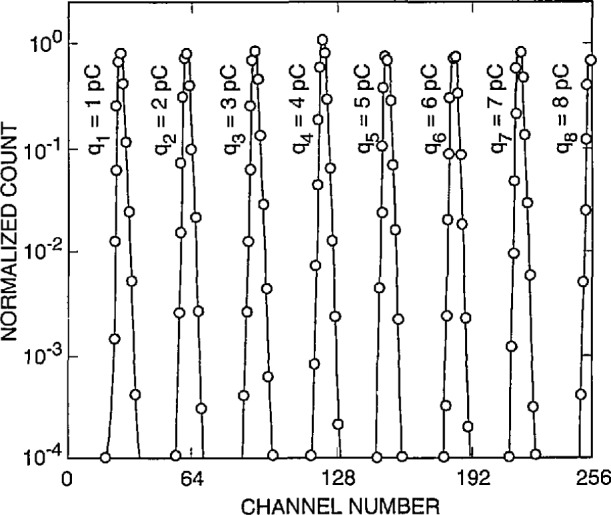
Amplitude-calibration data recorded by the MCA that show broadening due to noise.

The imposition of a constant background noise on the detected impulse signals introduces a broadening in the amplitude distributions recorded by the MCA. The broadening effect can be estimated from the convolution

$$P_{j}(\bar{q})=\int_{-\infty }^{+\infty }p_{j}(q^{\prime })f(\bar{q}-q^{\prime })dq^{\prime },$$ (26)

where $P_{j}(\bar{q})$ is the broadened distribution and $f(\bar{q}-q^{\prime })$ is a function that represents the statistical distribution about a mean value, $\bar{q}$, due to noise. In cases where the noise is inherent in the measurement system, the form of $f(\bar{q}-q^{\prime })$ can sometimes be estimated from the calibration data. Figure 24 shows an example of a set of calibration data recorded by the MCA under conditions where pulses of known amplitude, ${\bar{q}}_{i}(i=1,2,\ldots )$, are injected from the calibration source during fixed intervals of time. If noise were not present, counts would be recorded in only one channel of the MCA for each value of *qi.* The fact that counts appear in 12 or more channels for the data shown in Fig. 24 means that there is some broadening due to the presence of noise. In most cases like that shown in Fig. 24, the noise can be approximated by a Gaussian function, i.e.,

$$f(\bar{q}-q^{\prime })={\left(\pi w\right)}^{-\frac{1}{2}}\exp[-{\left(\bar{q}-q^{\prime }\right)}^{2}/w],$$ (27)

where the width, *w*, is independent of $\bar{q}$. Broadening due to noise can be significant if the condition Δ*q* ≫ *w* is not satisfied, where Δ*q* is the characteristic width *of p_j_*(*q*). If noise broadening is determined to be significant, then it may be possible to develop a deconvolution procedure using Eqs. (26) and (27) to obtain an improved estimate *of p_j_*(*q*) from the measured data recorded by the MCA. No attempts have been made to implement noise deconvolution procedures in the present work.

### 6.2 Amplitude Sum (Integrated-Charge) Distribution

The measurement of integrated-charge distribution, *p*_0_(*Q*^±^) for a specified phase region (positive or negative half-cycle) is subject to the same errors considered above for pulse-amplitude distribution, e.g., effects of amplifier nonlinearities and noise broadening. In addition to these, there are other mechanisms for introducing systematic errors that can result from the operating characteristics of the gated integrator and its associated input amplifier. These possible sources of error are examined here. Methods for calibration of the integrator that can reveal systematic errors and precautions that can be taken to ensure proper operation of this circuit are also considered.

It should first be realized that the output of the pulse amplifier (A2 in Fig. 4) is a rectangular pulse of constant width independent of the shape of the input pulse. The amplitude of the output pulse is directly proportional to the amplitude of the input pulse provided the input pulse lies above a critical value $q_{d}^{\prime }$ (discrimination level). The output of the amplifier, *q*, is related to the input, *q*′, by

$$q=g_{A2}q^{\prime },\;q^{\prime }>q_{d}^{\prime }$$ (28)

$$q=0,\;q^{\prime }<q_{d}^{\prime },$$ (29)

where *g*_A2_ is a constant corresponding to the gain of A2.

The input amplifier A2 essentially acts like a peak detector. By using this type of amplifier as an input to the integrator, the output pulse of the integrator is forced to be proportional in amplitude to the sum of the *amplitudes* of the pulses appearing at the input to A2 consistent with Eq. (4). The integrator, therefore, does not yield an output that is a true measure of the integrated current associated with the event pulses as given by

$$Q^{+}=\sum_{j}\int_{t-\tau_{j}}^{t+\tau_{j}}I_{j}^{\pm }(t^{\prime })dt^{\prime },$$ (30)

where $I_{j}^{\pm }(t^{\prime })$ is the instantaneous current of the *j* th pulse and *τ_j_* is the duration of the pulse defined such that $I_{j}^{\pm }(t^{\prime })\neq 0$ only for times in the interval *t* + *τ_j_* > *t*′ > *t* − *τ_j_.* The value of *Q*^±^ is directly proportional to that given by Eq. (4) only if all pulses have the same shape. The error in the determination of true integrated charge is approximately given by the difference

$$\Delta Q^{\pm }=\sum_{j}\left[q_{j}^{\pm }-\int_{t-\tau_{j}}^{t+\tau_{j}}I_{j}^{\pm }(t^{\prime })dt^{\prime }\right],$$ (31)

where the values for $q_{j}^{\pm }$ are determined by the calibration of amplitude in terms of charge-per-pulse under conditions where pulse shape is governed by the detector impulse response (see previous section).

For cases where there may be events having pulse durations, *τ_j_*, that exceed the width of the impulse response, the sign of the error Δ*Q*^±^ is most likely negative, i.e., the measurement preferentially tends to underestimate the integrated charge. An additional contribution to a negative error can occur if there are events with amplitudes lower than the discrimination level $q_{d}^{\prime }$ defined in Eq. (28). The integrated charge for these events is simply not included in the sum of amplitudes, Eq. (4).

**Fig. 25:**
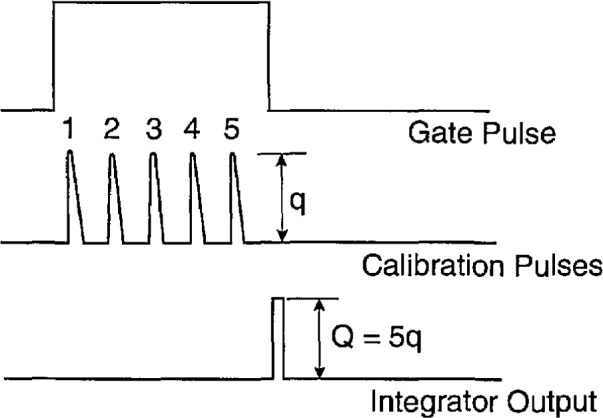
Pulse timing diagram that applies to a calibration of the gated integrator. The output of the integrator should ideally be a pulse with an amplitude directly proportional to the sum of the amplitudes of the cahbration pulses that occur during the gate pulse.

Calibration of the integrator requires the use of a gated pulse generator that produces a burst of pulses only during the time that the integrator is gated on as shown in Fig. 25. If *N*_I_ calibration pulses of known amplitude *qc* are applied during the gate interval, then the output pulse of the integrator should ideally have an amplitude directly proportional to the product *N*_I_*q*_c_. This means that the output should be independent of the number of pulses used if *q*_c_ is maintained at a value of *V*_c_*/N*_I_ where *V*_c_ is constant. Thus, if *N*_I_ is increased by a factor of two, the integrator should give the same output provided *q*_c_ is correspondingly reduced by a factor of one half.

To ensure proper operation of the integrator, the dc bias level at the output of A2 should be adjusted to zero. After this adjustment is made, the offset voltage of the integrator (amplifier Al in Fig. 18) should be set at a value that forces the integrator output pulse amplitude to be zero when no pulses are applied to the input, i.e, when *N*_I_ = 0. This latter adjustment is required because the 0.01 μF integrating capacitor in Fig. 18 can acquire an initial small charge attributable to the transient voltage associated with the opening of the FET’s T1 and T2. Failure to make these adjustments will allow systematic errors to occur in the measured integrated charge due to an offset, i.e., a nonzero intercept of the calibration curve.

**Fig. 26:**
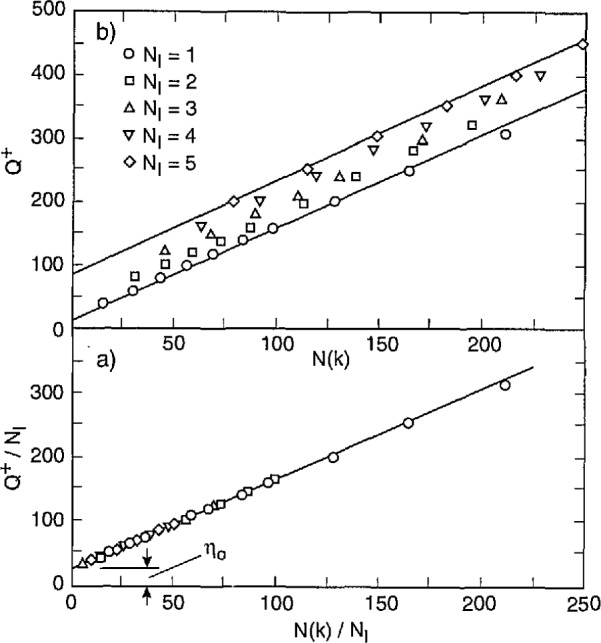
Example of an integrator calibration performed under conditions where there is a finite offset corresponding to a non-zero intercept *η*_0_ as shown in a). The calibration data in b) indicate the resulting uncertainty due to this offset (range of *Q^+^* defined by the solid lines).

**Fig. 27:**
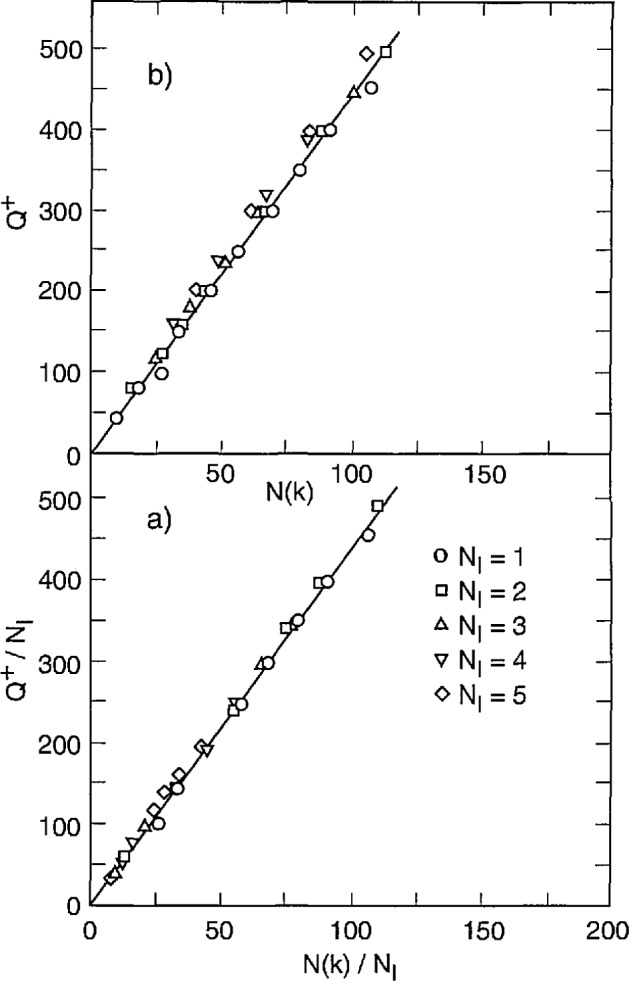
Example of an integrator calibration performed under conditions of zero offset (zero intercept, *η*_0_) as shown in a); and corresponding error reduction as seen in b).

Errors introduced by the presence of a finite offset are illustrated by the examples of calibration data shown in Figs. 26 and 27. Plotted in Fig. 26a is the amplitude (or equivalently charge) per calibration pulse, *q*_c_ = *Q*^±^/*N*_I_, versus the recorded MCA channel number per pulse, *N*(*k*), for *N*_I_ = 1 to 5. Figure 26 shows the corresponding calibration plot of *Q*^±^ versus *N*(*k*), again for *N*_I_ = 1 to 5. The data in Fig. 26a tend to fall on a straight line given by

$$Q^{\pm }/N_{I}=\eta_{0}+\eta_{1}N(k)/N_{I},$$ (32)

where *η*_1_ is the slope and *η*_0_ is the intercept corresponding to a constant offset voltage. The corresponding integrator calibration curves are given by

$$Q^{\pm }=N_{I}\eta_{0}+\eta_{1}N(k).$$ (33)

Consistent with the data shown in Fig. 26b, the intercepts for the *Q*^±^ versus *N*(*k*) curves increase with increasing *N*_I_. Under these operating conditions, there is an uncertainty of *η*_0_Δ*N*_I_ in the amplitude sum (integrated charge) due to the off-set, where Δ*N*_I_ is given by

$$\Delta N_{I}=N_{I}(max)-N_{I}(min).$$ (34)

Here, *N*_I_(max) and *N*_I_(min) are respectively the maximum and minimum number of events that are likely to be recorded within the integrator gate interval (Δ*t*_I_ in Fig. 19). The solid lines in Fig. 26a represent the error limits for the case considered when *N*_I_(max) = 5 and *N*_I_(min) = 1. Figure 27 shows how this systematic error can be reduced if care is taken to minimize the offset so that *η*_0_≈0.0. In general, all stages of amplification should be adjusted individually to eliminate offsets. Further reduction in the error can be achieved by giving the greatest weight to calibrations made using values for *N*_I_ that equal the mean number of experimentally observed events within the integrating interval.

### 6.3 Time-Interval and Phase-of-Occurrence Distributions

**Fig. 28:**
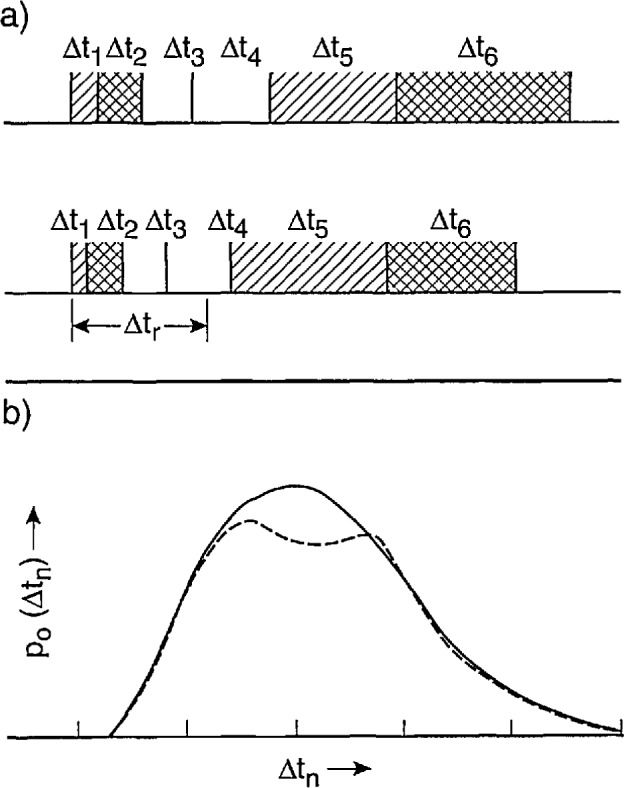
Possible distortion of a measured time-separation distribution, *p*_0_(Δ*t_n_*), for pulse bursts due to the finite TAG reset time Δ*t_r_.*

Systematic errors that can occur in the measurement of pulse time-separation distributions were analyzed in our earlier work [24]. It was noted that the measured time-separation distributions can become distorted if: a) a significant fraction of the time separations are less than the time-to-amplitude converter reset time, Δ*t*_r_, and b) there are correlations among successive time separations. The reason why distortions are introduced under these conditions can be understood from a consideration of the example illustrated in Fig. 28. It is assumed that the phenomenon of interest appears in the form of pulse bursts where there is an ordering of pulse time separations within a burst such that the first separation is on the average smaller than the second and so on. This type of behavior occurs, for example, in the case of burst-type positive-corona pulses generated in sulfur hexafluoride using point-plane electrode gaps [27].

Figure 28a shows pulse diagrams for two different bursts of 7 pulses each with the indicated successive time separations Δ*t*_1_, Δ*t*_2_,… and the TAC reset time Δ*t*_r_. It is assumed that two TACs are used for the measurement as shown in Fig. 3. The shaded time separations are those actually recorded by the system, i.e., Δ*t*_1_, and Δ*t*_5_ are measured by TAC1 and Δ*t*_2_ and Δ*t*_6_ are measured by TAC2. The time separations Δ*t*_3_ and Δ*t*_4_ are not recorded because they occur before the TAC has had time to reset. The failure to record these separations causes the measured distribution to deviate from the true distribution as shown in Fig. 28b. This limitation can be overcome to some extent by using multiple TACs as discussed in the next section.

**Fig. 29:**
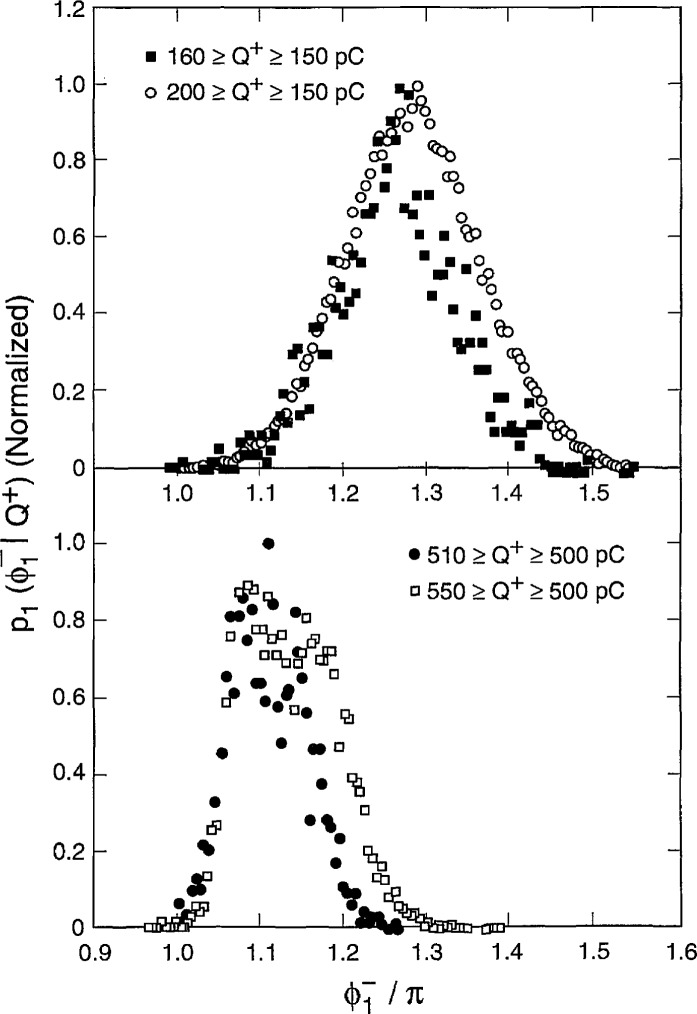
Examples of data for measured conditional phase-of-occurrence distributions $p_{1}(\phi_{1}^{-}|Q^{+})$ that show asymmetric broadening due to a finite window for the “fixed” variable *Q*^+^.

In the case of conditional time-interval or phase- of-occurrence distributions, distortions can also occur if the range of values for the “fixed” variable are not sufficiently well restricted. As noted above [see Eq. (9)], the necessity of using a finite window size for the fixed variable introduces a broadening of the distribution. The problem has already been noted for the measurement of conditional pulse-amplitude distributions [24]. Unlike the broadening due to noise, the broadening introduced by a finite window can be asymmetrical with a resultant apparent shift in the associated mean value. Examples of asymmetric broadening due to an increase in window for the variable *Q*^±^ are shown in Fig. 29 for measured conditional distributions $p_{1}(\phi_{1}^{-}|Q^{+})$ corresponding to the phase of the first negative PD pulse generated in a point-dielectric discharge gap. In general, it is desirable to keep the window size to the minimum required to obtain acceptable statistics within a reasonable time. Excessive distortions due to finite window size will invalidate consistency analysis using the various integral relationships among measured conditional and unconditional distributions, e.g., using Eqs. (7), (8), (13), and (14).

## 7. Limitations, Extensions, and Alternatives

### 7.1 Limitations

It was noted at the outset that the system described in this work is optimally designed to investigate the stochastic properties of ac or dc generated PD pulses with repetition rates between 50 and 5 × 10^4^/s. The lower limit on pulse rate is determined by the acceptable times within which observations can be made that will yield statistically significant results. If the phenomenon of interest is stationary over the time of observation, then there is, in principle, no lower limit on the pulse rate that could be observed. However, if the time between pulses exceeds the range of a TAC (typically 1 s), then time intervals must be measured by another method, e.g., using a digital clock with a gated pulse counter. For extremely low pulse rates (less than 1 per min) it may be more efficient to simply perform a statistical analysis of recorded data.

The upper limit on the rate of observed pulses is imposed by time restrictions inherent to the electronics. Specifically, there are limits due to: 1) the finite reset time of the TAC, 2) the dead-time of the MCA, 3) built-in delays in the SCA and Δ*t* control logic circuits, and 4) the inherent impulse response of the pulse detection and amplifier network. It may be possible by using a broad-band detector and faster electronics to increase the range of applicability by an order-of-magnitude, i.e., to 5×10^5^/s. However, this may introduce added complexity and cost that would make alternative methods appear more attractive such as the “software” approach considered in Sec. 7.3.

Although it was assumed here that pulse amplitude (or the sum of successive pulse amplitudes) is the appropriate “mark” for characterizing the intensity of the phenomenon, there may exist cases where other marks such as pulse area or pulse-shape parameters are more appropriate indicators of “intensity.” The system documented here may still be applicable to these cases provided the mark can be converted to a pulse with an amplitude that is directly proportional to the “size” of the mark.

In principle, there is no upper limit to the size of a mark that can be measured. In some cases it may be necessary to restrict the amplitude of an event pulse by a linear attenuation network so that it does not exceed the voltage range acceptable to the MCA (0 to 8V in the present system). If the dynamic range of pulse amplitudes is very large (two or more orders-of-magnitude), then it may be necessary or desirable to replace the linear input pulse amplifier with a logarithmic amplifier.

The lower limit on acceptable pulse amplitude is simply governed by signal-to-noise ratio. As the broadening due to noise (see Sec. 6.1.3) becomes comparable to the width of the observed distribution, it becomes increasingly difficult to extract meaningful information about memory effects from the data.

It was noted previously that when broadening due to noise or restricted variable window size is significant, it may no longer be possible to perform a consistency analysis among various measured distributions using the integral relationships that connect these distributions, e.g., Eq. (7). It may still be possible, nevertheless, to use the data on conditional distributions to establish the *existence* of memory propagation. The existence of memory propagation can be unequivocally established if it can be shown, for example, that the conditional distributions *p*_1_(*q_j_*|Δ*t_j_*_−1_ ϵ (Δ*t_a_*, Δ*t*_b_)) and $p_{1}(q_{j}|\Delta t_{j-1}\epsilon (\Delta t_{a^{\prime }},\Delta t_{b^{\prime }}))$ do not coincide under at least one condition where the corresponding ranges of the time intervals (Δ*t_a_*, Δ*t*_b_) and $\left(\Delta t_{a^{\prime }},\Delta t_{b^{\prime }}\right)$ are different. In order to determine this lack of coincidence, it is necessary: 1) to acquire enough data to demonstrate a statistically significant difference between the two distributions, and 2) to acquire the data under conditions where the phenomenon is stationary. The effects of non-stationary behavior can be minimized if the data for the two conditional distributions can be accumulated simultaneously. Unfortunately, in the present system, which has only one Δ*t* control logic circuit and one MCA, it is not possible to make simultaneous measurements of two distributions of the same type. The system can be operated, however, to alternately accumulate data in two different 256-channel segments of the 1024-channel MCA for two different ranges of the fixed variable. By periodic switching back and forth between the two segments, it may be possible to “average out” effects of nonstationary behavior. The existence of nonstationary behavior can often be detected from periodic monitoring of unconditional distributions such as *p*_0_(*q_j_*) or *p*_0_(*Q*^±^) for which statistically significant data can be accumulated in much shorter times than for conditional distributions for which pulse count rates are lower. It has been shown [1,4] that the profiles of unconditional distributions tend to be more sensitive to nonstationary behavior than the profiles of conditional distributions.

It should be realized that, in general, the time required to obtain statistically significant data for a distribution can increase rapidly as the number of restrictions imposed by the fixed variables is increased and as their ranges are reduced. This is perhaps the most stringent limitation inherent not only to this measurement system but also to stochastic analysis in general. For the types of pulsating PD phenomena investigated with this system, it has usually not been possible to obtain enough data with adequate statistics for conditional distributions higher than second order.

### 7.2 Extensions

Some of the limitations mentioned in the previous and earlier sections can be overcome (or at least reduced) by introducing various extensions or expansions to the existing measurement system. One obvious extension would be to introduce additional MCAs with associated circuitry to allow simultaneous measurement of two or more distributions. This would not only reduce the total data acquisition time, but would also allow better monitoring of effects due to nonstationary behavior. Since nonstationary behavior in PD phenomena is often a consequence of discharge-induced modifications of the discharge gap, e.g., changes in rates of electron emission from surfaces, multiple MCA measurement capability might make the system more useful as a diagnostic of insulation aging.

The previously noted limitations on the measurement of time or phase separation distributions imposed by the finite TAC reset time can be overcome by incorporating more TACs in series with an associated pulse sorting circuit so that each TAC measures a different successive time interval. If instead of using only two TACs one uses 2 *n* TACs, where *n* > 2, then the minimum time separation that can be measured without introducing errors (see Sec. 6.3) is reduced from Δ*t*_r_/2 to Δ*t*_r_/2*n.*

Of course, the introduction of added instrumentation such as MCAs and TACs significantly increases the cost of the system. In some cases this added cost may be more than compensated for by the reduction in time required to acquire and analyze the data. Additional time savings may be achieved by automating the system to allow not only the simultaneous measurement of more than one distribution but also real-time calibration, data analysis, and optimization of time spent where needed to obtain the best statistics.

Finally, it should be noted that although the system described here has only been applied to the measurement of pulse sequences in real time, it is also possible to use it for stochastic analysis of pre-recorded pulses. This merely requires that the source of the input pulses be derived from an electronic recording device operated in the “play-back” mode. As will be argued in the next section, it should be possible to use prerecorded data derived from computer simulations to test the overall system performance. It is, of course, desirable that the simulation produce pulses with known stochastic properties, i.e., conditional distributions that mimic those of the phenomena under investigation, and that simulated pulses have an amplitude and shape similar to experimentally observed pulses. The use of the system described here to perform analysis on prerecorded data allows an obvious extension to pulses with repetition rates that are higher or lower than the ranges which are normally acceptable. However, one should consider whether or not it may be more efficient to analyze prerecorded data directly using computer software such as considered in the next section.

### 7.3 Alternatives

One of the primary advantages of the system described in this work is the ability to measure the stochastic properties of a pulsating phenomenon in real time. It allows one to view on a computer output device (video monitor) the development of conditional or unconditional distributions as the data are acquired. With this capability, it is possible for the operator to determine quickly the existence of memory effects and to make decisions on the conditions that should be selected to yield the most interesting data.

The system, in its present form, is designed to be a *research tool* for use in investigating memory propagation in pulsating phenomena. It is assumed that it is operated by those who have a thorough understanding of the phenomenon under investigation. Because this is a highly “interactive” system, the quality of the information acquired from the measurements will be determined to a large extent from the judgments of the operator. It may be possible to construct an “automated” real-time stochastic analyzer based on the measurement concepts introduced here. Some aspects of the present system could, for example, be incorporated into advanced partial-discharge measurement systems that would allow the possibility for meaningful pattern recognition needed to identify types of discharge sites.

Another advantage of a real-time measurement system is that it overcomes problems of storing large data files. For example, the measurement of some of the second-order distributions shown in Figs. 22 and 23 required ten or more minutes of data acquisition time. This means that only a very small fraction of the total number of discharge events that occurred during the measurement time were actually recorded. A record of all discharge events that occurred in a typical 10 min segment would generally contain data for more than 10^9^ pairs of numbers. Within the times required to obtain reasonably good statistics on higher-order conditional distributions, it is possible to generate data files containing all events that exceed minicomputer storage capacity. Analysis could then only be performed using either “main-frame” type computers or appropriately segmented data files in smaller computers.

The obvious disadvantage of the present measurement system is that it does not make efficient use of the available data. In the measurement of conditional distributions, most of the information about the impulse events is discarded. Once the data are discarded, they can no longer be retrieved for subsequent analysis. The expense of introducing additional MCAs in parallel to enhance the information retrieval efficiency can make the cost of the system prohibitive.

In cases where either the amount of data is severely limited or the phenomenon is highly non-stationary, it may be essential to consider all of the available data. There may also be other cases where it is necessary to work with prerecorded data due to externally imposed geometrical or time constraints.

The most efficient use of available data in such cases can, at least in principle, be achieved using an alternate approach that places more reliance on computer software. An example is given below of an algorithm that was developed to acquire data on conditional and unconditional distributions from a Monte Carlo simulation of ac-generated partial discharges. Details of the theoretical model upon which the simulation is based are given elsewhere [33] and will not be covered here. It need only be said that the simulation produces a sequence of phase-correlated pulses with stochastic properties similar to those observed for PD generated by applying an alternating voltage to a point-dielectric electrode configuration [3].

A partial listing of a FORTRAN-77 routine used to sort data in real-time for determination of the distributions that apply to simulated PD is given in Table 6. The specific distributions considered in this table are the unconditional and conditional phase-of-occurrence distributions for the *i* th negative PD pulse, i.e., $p_{0}(\phi_{i}^{-})$ and $p_{1}(\phi_{i}^{-}|Q^{+})$.

**Fig. 30:**
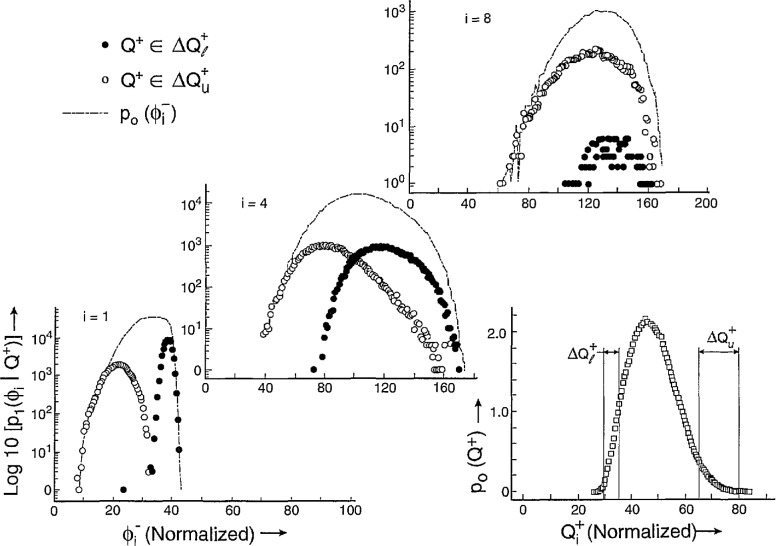
Examples of results from a computer analysis of PD pulses generated using a Monte Carlo simulation. Shown are the conditional and unconditional distributions determined from a data sorting routine similar to that listed in Table 6.

The first statement in this routine converts the value of the normalized phase $\left(\phi_{i}^{-}/2\pi \right)$ to the nearest integer value between 0 and 200. It thus performs essentially the same discretization of the data as in a 200-channel MCA. The assigned integer value is then used to identify elements of three-dimensional integer arrays corresponding to particular distributions. In the case of the unconditional phase distributions, the array element is increased by 1, i.e., by one pulse count. In the case of the conditional distributions, the array elements associated with $p_{I}(\phi_{i}^{-}|Q_{U}^{+})$ and $p_{I}(\phi_{i}^{-}|Q_{L}^{+})$ are incremented by 1 only if the values of $Q_{U}^{+}$ and $Q_{L}^{+}$ lie within specified ranges. An example of the results from this routine are shown in Fig. 30 together with the corresponding data for *p*_0_(*Q^+^*) that indicate the ranges selected for $Q_{U}^{+}$ and $Q_{L}^{+}$. It is seen that the conditional distributions from this simulation show the same stochastic trends seen for the experimental data in Fig. 22, i.e., $\left(Q^{+}↑\Rightarrow \phi_{i}^{-}↓\right)$ for all values of *i*.

The routine shown in Table 6 is used to analyze simulated PD pulses in real time. The value for *Q^+^* is stored from the previous half-cycle. Each time a negative pulse is generated, its number, phase, and amplitude are tested and selected for inclusion in various bins associated with distributions such as considered in Table 6. Data for many different distributions can be simultaneously recorded in this way. Thus it is possible using software to make efficient use of available data without generating large intermediate data files.

In order to implement this approach in a measurement system, it is necessary that the data be converted to digitized pairs of numbers corresponding to pulse amplitude and time (or phase) that can be accessed sequentially by a computer. Such a system could, of course, also be used to analyze prerecorded data. Although this approach appears feasible using existing analog-to-digital conversion methods and computer technology, no attempts have been made to develop the required hardware.

It should finally be noted that Monte Carlo simulations such as the one that yielded the results shown in Fig. 30 could be used to test the performance of stochastic analyzers. This can be accomplished by using a digital-to-analog converter to produce a sequence of pulses from the output of the computer simulation. The conditional and unconditional distributions measured for the simulated pulses can be compared with the known distributions determined from an analysis made using computer routines such as given in Table 6. The results from the simulation can also be used to provide an indication of the quantities of data needed to obtain statistically meaningful results. The results shown in Fig. 30 correspond to 10^6^ cycles of the excitation voltage. It is clear that the quantity of data for $p_{I}(\phi_{8}^{-}|Q^{+})$ is close to the minimum needed for determination of a statistically significant memory effect.

The possibilities thus appear to exist for constructing efficient computer-based stochastic analyzers that can essentially duplicate and extend the capabilities of the system described here. There are no reasons why such a system could not be designed to accept either analog or digital data directly from measuring devices or indirectly from prerecorded files. The correct performance of the system can be verified using reference data with known stochastic behavior such as generated with a Monte Carlo simulation.

## Figures and Tables

**Table 1:** Measurable conditional and unconditional pulse-amplitude and time-separation distributions for a constant excitation process (de-generated PD)

Distribution type	Amplitude distribution	Time-separation distribution
Unconditional	*p*_0_(*q_j_*)	*p*_0_(Δ*t_j_*)
First-order conditional	*p*_1_(*q_j_*|Δ*t_j_*_−1_)	*p*_1_(Δ*t_j_*|Δ*t_j_*_−1_)
	*p*_1_(*q_j_*|*q_j_*_−1_)	*p*_1_(Δ*t_j_*|*q_j_*)
	*p*_1_(*q_j_*|Δ*t_j_*_−_*_k_*),*k* > 1	
Second-order conditional	*p*_2_(*q_j_*|Δ*t_j_*_−1_,*q_j_*_−1_)	
	*p*_2_(*q_j_*|Δ*t_j_*_−1_,Δ*t_j_*_−2_)	

**Table 2:** Measurable conditional and unconditional pulse-amplitude and phase distributions for a periodic, time varying excitation process (ac-generated PD)

Distribution type	Amplitude distribution	Phase distribution	Pulse number and cycle specifications
Unconditional (unspecified event)	$p_{0}(q_{l}^{\pm })$	$p_{0}(\phi_{l}^{\pm })$	all, *i* ⩾ 1
Unconditional (specified event)	$p_{0}(q_{l}^{\pm })$	$p_{0}(\phi_{i}^{\pm })$	*i* = 1,2,3,…
First-order conditional	$p_{1}(q_{l}^{\pm }|\Delta \phi^{\pm })$		$\phi_{l}^{\pm }\epsilon \Delta \phi^{\pm }$, all, *i* ⩾ 1
	$p_{1}(q_{l}^{\pm }|\phi_{i}^{\pm })$	$p_{1}(\phi_{i}^{\pm }|\phi_{i-1}^{\pm })$	*i* = 1,2,3…
	$p_{1}(q_{ij}^{\pm }|Q_{k}^{\pm })$	$p_{1}(\phi_{ij}^{\pm }|Q_{k}^{\pm })$	*i* = 1,2,3…
			+ *→ k* = *j*, *j*−l,…
			− → *k* = *j*−1, *j*−2,…
	$p_{1}(q_{l}^{\pm }|\Delta \phi_{l.l-1}^{\pm })$		*i* = 2,3,4,…
Second-order conditional	$p_{2}(q_{l}^{\pm }|\phi_{l-1}^{\pm },q_{l-1}^{\pm })$	$p_{2}(\phi_{l}^{\pm }|\phi_{l-1}^{\pm },q_{i-1}^{\pm })$	*i* = 2,3,4,… or all *i* ⩾ 2
	$p_{2}(q_{ij}^{\pm }|\phi_{ij}^{\pm },Q_{k}^{\pm })$		*i* = 1,2,3,…
			*+* → *k* = *j*, *j*−1,…
			− → *k* =*j*−1, *j*−2,…
Third-order conditional	$p_{3}(q_{i}^{\pm }|\phi_{i}^{\pm },q_{l-1}^{\pm },\phi_{i-1}^{\pm })$	$p_{3}(\phi_{ij}^{\pm }|\phi_{l-1,j}^{\pm },q_{i-1,j}^{\pm },Q_{k}^{\pm })$	*i* = 2,3,4…
			*+* → *k* = *j*, *j*−1,…
			− → *k* = *j*−1, *j*−2,…

**Table 3:** Configuration of switch connections for the system shown in Fig. 3 that are required for measurement of the various conditional and unconditional pulse-amplitude and time-interval distributions for a constant excitation process

Distribution	Switch						
	S1	S2	S3	S4	S5	S6	S7
*p*_0_(*q_j_*)	x1=z1	*	*	x4=y4	*	*	x7=z7
*p*_1_(*q_j_*|Δ*t_j_*_−1_)	x1=z1	x2=y2	x3 = w3	x4 = x4	*	*	x7=z7
*p*_1_(*q_j_*|Δ*t_j_*_−1_), *k*>1	x1=z1	x2=y2	x3 = w3	x4 = z4	*	*	x7=z7
*p*_2_(*q_j_*|Δ*t_j_*_−1_,*q_j_*_−1_)	x1=z1	x2 = z2	x3 = z3	x4=w4	*	*	x7=z7
*p*_2_(*q_j_*|Δ*t_j_*_−1_,Δ*t_j_*_−2_)	x1=z1	x2 = z2	x3=y3	x4 = w4	*	*	x7=z7
*p*_0_(Δ*t_j_*)	x1=y1	*	*	*	*	x6 = z6	x7=y7
*p*_1_(Δ*t_j_*|Δ*t_j_*_−1_)	x1=y1	*	*	*	x5 = z5	x6=y6	x7=y7
*p*_1_(Δ*t_j_*|Δ*q_j_*)	x1=y1	*	*	*	x5=y5	x6=y6	x7=y7

* Switch position irrelevant.

**Table 4:** Configuration of switch connections for the system shown in Fig. 4 required for measurement of the various conditional and unconditional amplitude or total charge distributions for a periodic time-varying excitation process

Distribution	Switch					
	S1	S2	S3	S4	S5	S6
$p_{0}(q_{i}^{\pm })$, *i* ⩾1	*	*	x3 = y3	x4 = z4	*	x6 = z6
$p_{0}(q_{1}^{\pm })$	x1 = z1	x2 = y2	x3 = w3	x4 = y4	x5 = z5	*
$p_{0}(q_{i}^{\pm })$, *i* = 2,3,…	x1 = z1	x2 = y2	x3 = w3	x4 = y4	x5 = w5	*
$p_{1}(q_{i}^{\pm }|\Delta \phi^{\pm })$	*	*	x3 = w3	x4 = z4	*	x6 = z6
$p_{1}(q_{1}^{\pm }|\phi_{1}^{\pm })$	x1 = z1	x2 = y2	x3=w3	x4=w4	x5 = z5	*
$p_{1}(q_{i}^{\pm }|\phi_{i}^{\pm })$, *i* = 2,3,…	x1 = z1	x2 = y2	x3 = w3	x4 = w4	x5 = w5	*
$p_{1}(q_{1}^{\pm }|Q^{\mp })$	x1 = z1	x2 = z2	x3 = w3	x4 = y4	x5 = z5	*
$p_{1}(q_{i}^{\pm }|Q^{\mp })$, *i* = 2,3,…	x1 = z1	x2 = z2	x3 = w3	x4 = y4	x5 = w5	*
$p_{2}(q_{1}^{\pm }|\phi_{1}^{\pm },Q^{\mp })$	x1 = z1	x2 = z2	x3 = w3	x4 = y4	x5 = z5	*
	x1 = z1	x2 = z2	x3 = w3	x4 = y4	x5 = z5	*
$p_{2}(q_{i}^{\pm }|\phi_{i}^{\pm },Q^{\mp })$, *i* = 2,3,…	x1 = z1	x2 = z2	x3=w3	x4 = w4	x5=w5	*
$p_{1}(q_{i}^{\pm }|\Delta \phi_{i,i-1}^{\pm })$, *i* = 2,3,…	x1=z1	x2 = y2	x3 = w3	x4 = y4	x5 = w5	*
$p_{2}(q_{i}^{\pm }|\phi_{i-1}^{\pm },q_{i-1}^{\pm })$, *i* ⩾ 2	*	*	x3 = w3	x4 = y4	x5 = y5	*
$p_{3}(q_{i}^{\pm }|\phi_{i}^{\pm },\phi_{i-1}^{\pm }q_{i-1}^{\pm })$, *i* ⩾ 2	*	*	x3 = w3	x4 = y4	x5 = y5	*
*p*_0_(*Q*^∓^)	*	*	x3 = z3	x4 = z4	*	x6 = y6

* Switch position irrelevant.

**Table 5:** Configuration of switch connections for the system shown in Fig. 4 required for measurement of conditional phase distributions for a periodic, time-varying excitation process

Distribution	Switch					
	S1	S2	S3	S4	S5	S6
$p_{0}(\phi_{i}^{\pm })$, *i* = 1.2…	x1 = z1	x2 = y2	x3=y3	x4 = z4	*	x6 = y6
$p_{1}(\phi_{i}^{\pm }|Q^{\mp })$, *i* = 1.2,…	x1 = z1	x2 = z2	x3 = y3	x4 = z4	*	x6 = y6
$p_{1}(\phi_{i}^{\pm }|\phi_{i-1.}^{\pm })$, *i* = 2,3…	x1=y1	x2 = y2	x3 = y3	x4 = x4	x5 = y5	x6 = y6
$p_{2}(\phi_{i}^{\pm }|\phi_{i-1}^{\pm },q_{i-1}^{\pm })$, *i* = 2,3…	x1=y1	x2 = y2	x3 = y3	x4 = z4	x5 = y5	x6 = y6
$p_{3}(\phi_{i}^{\pm }|\phi_{i-1}^{\pm },q_{i-1}^{\pm },Q^{\mp })$, *i* =2,3,…	x1 = y1	x2 = z2	x3=y3	x4 = z4	x5 = y5	x6=y6

* Switch position irrelevant.

**Table 6:** Partial listing of a FORTRAN 77 routine for determining the distributions $p_{0}(\phi_{i}^{-})$ *and* $p_{1}(\phi_{i}^{-}|Q^{+})$ from numerical data on $\phi_{i}^{-}$ and two windows for *Q^+^*, i.e., $Q^{+}\epsilon (Q_{1U}^{+},Q_{1L}^{+})$ and $Q^{+}\epsilon (Q_{2U}^{+},Q_{2L}^{+})$.

C	POPI = ARRAY FOR UNCONDITIONAL PHASE DIST.
C	PIPI1, PIPI2 = ARRAYS FOR CONDITIONAL PHASE DIST.
C	QPOS = INTEGRATED POSITIVE CHARGE (AMPLITUDE SUM).
C	QPU1, QPL1 = LIMITS ON WINDOW-1 FOR QPOS.
C	QPU2, QPL2 = LIMITS ON WINDOW-2 FOR QPOS.
C	IN = NEGATIVE PULSE INDEX NUMBER.
C	CHANNEL = INTEGER BETWEEN 0 AND 200.
C	PHASE = PHASE OF NEGATIVE PULSE.
	CHANNEL = NINT (200^*^PHASE)
	POPI (CHANNEL, IN, 0) = POPI (CHANNEL, IN, U) + I
	IF(QPOS. LT. QPU1) THEN
	IF(QPOS. GT. QPL1) THEN
	PIPI1 (CHANNEL, IN,, 0) = PIPI1 (CHANNEL, IN, 0) + I
	END IF
	END IF
	IF (QPOS. LT. QPU2) THEN
	IF (QPOS. GT. QPL2) THEN
	PIPI2 (CHANNEL,IN, 0) = PIPI2 (CHANNEL, IN, 0) + 1
	END IF
	END IF
